# Characterization of *aliA* and *aliB* deletion mutants reveals a dominant role of AliA in *Haloferax volcanii* lipoprotein lipidation

**DOI:** 10.1016/j.jbc.2026.113477

**Published:** 2026-08-25

**Authors:** Yirui Hong, Andy Garcia, Samuel Kopelev, Jacob A. Cote, Friedhelm Pfeiffer, Paula Welander, Stefan Schulze, Mechthild Pohlschroder

**Affiliations:** 1 Department of Biology, University of Pennsylvania, Philadelphia, Pennsylvania, USA; 2 Department of Earth System Science, Stanford University, Stanford, California, USA; 3 Thomas H. Gosnell School of Life Sciences, College of Science, Rochester Institute of Technology, Rochester, New York, USA; 4 Computational Systems Biochemistry, Max Planck Institute of Biochemistry, Martinsried, Germany

**Keywords:** archaea, *Haloferax volcanii*, cell surface, lipoprotein, prenylation, proteomics

## Abstract

Protein lipidation is a widespread strategy for anchoring proteins to cellular membranes across all domains of life, yet the mechanisms underlying this process in archaea remain poorly understood. Recently, the first archaeal enzymes involved in lipobox-containing protein (lipoprotein) biogenesis, AliA and AliB, were identified and characterized in the model archaeon *Haloferax volcanii*. Although these paralogs share significant sequence similarity, distinct deletion phenotypes suggest differences in their substrate specificity and function. Here, we employed proteome-wide Triton X-114 fractionation followed by quantitative proteomics and lipid-specific mass spectrometry to systematically analyze AliA- and AliB-dependent lipoprotein lipidation. Deletion of *aliA* affected substantially more lipoproteins in *Hfx. volcanii* than deletion of *aliB*, markedly diminishing their TX-114 enrichment—indicating reduced hydrophobicity—and abolishing thioether-linked archaeol modification. This establishes AliA as the primary enzyme responsible for archaeal lipoprotein lipidation. In contrast, deletion of *aliB* affected only a small subset of lipoproteins and did not reduce thioether-linked archaeol levels. In addition to defining distinct and non-redundant roles for AliA and AliB, this study provides the first large-scale experimental validation of predicted archaeal lipoproteins and identifies candidate components of the archaeal lipoprotein biogenesis pathway, substantially advancing mechanistic understanding and enabling improved lipoprotein prediction in this previously underexplored field.

Protein lipidation is a widely conserved phenomenon observed across all domains of life (1, 2, 3, 4). It substantially increases the hydrophobicity of proteins, thereby influencing their structures, stability, subcellular localization, enzymatic activities, and interactions with membranes and other proteins. Extensive studies in eukaryotes and bacteria have revealed a highly diverse landscape of lipidation types, each mediated by distinct enzymes and characterized by different conserved motifs, lipid anchors, biochemical properties, and biological functions (1, 2, 3, 5). In Archaea, a domain of life with important implications for eukaryogenesis (6, 7, 8), ecology (9, 10), and biotechnology (11, 12), protein lipidation exhibits distinct features compared to the other two domains. While two lipidation types commonly seen in eukaryotes and/or bacteria, GPI anchoring (13) and acylation (14, 15, 16), have also been reported in archaea, the predominant form of archaeal protein lipidation is expected to be prenylation (17, 18, 19, 20, 21, 22, 23, 24), the addition of isoprenoid-derived moieties to proteins. This preference is likely attributed to the unique composition of archaeal membrane lipids, which are composed predominantly of isoprenoid-based alkyl chains ether-linked to glycerol-1-phosphate backbones (25, 26, 27), in contrast to the fatty acyl chains ester-linked to glycerol-3-phosphate backbones in eukaryotes and most bacteria (28, 29, 30).

Two major classes of prenylated proteins have been reported in archaea: the C-terminally lipidated ArtA (archaeasortase A) substrates (20, 21, 23, 24, 31) and N-terminally lipidated proteins that contain a signature lipobox motif ([L/V/I]^−3^ [A/S/T/V/I]^−2^ [G/A/S]^−1^ [C]^+1^) (4, 32). These lipobox-containing proteins are also widespread in bacteria and are commonly referred to as bacterial lipoproteins in the literature (33, 34). In bacteria, lipoprotein biogenesis begins with the transport of lipoprotein precursors to cell membranes either in an unfolded state *via* the general secretory (Sec) pathway or in a folded state *via* the twin-arginine translocation (Tat) pathway (35). The maturation of bacterial lipoproteins is subsequently carried out through a highly conserved three-step process. First, prolipoprotein diacylglyceryl transferase (Lgt) catalyzes the covalent attachment of a membrane lipid moiety to the lipobox cysteine (36). Next, the lipoprotein signal peptidase (Lsp) cleaves the signal peptide N-terminal to the modified cysteine (37). Finally, in some lipoproteins, the α-amino group of the cysteine undergoes further lipidation by lipoprotein N-acyltransferase (Lnt) (38), lipoprotein *N*-acyl transferase system A and B (LnsAB) (39), or lipoprotein intramolecular transacylase (Lit) (40). A substantial fraction of archaeal proteins contain a canonical lipobox motif with an essential cysteine and are therefore considered to be archaeal lipoproteins (4, 41). However, despite this widespread genomic signature, only a small number of predicted archaeal lipoproteins have been experimentally confirmed (4, 42, 43, 44). Moreover, no homologs of the bacterial lipoprotein biosynthetic enzymes have been reported in archaea, consistent with the substantial differences in membrane lipid composition between the two domains.

Recently, by combining bioinformatic analyses with biochemical characterization, we identified two paralogous proteins, AliA and AliB, as the first known components of the archaeal lipoprotein biogenesis pathway (43). While AliB is restricted to Haloarchaea, AliA is widely conserved across archaeal species. Deletion of *aliA* and/or *aliB* in the model archaeon *Haloferax volcanii* impaired lipoprotein biogenesis and caused diverse physiological defects, including slower growth, decreased motility, and altered cell shape. Deletion of both genes resulted in a complete loss of thioether-linked archaeol modification in lipoprotein extracts, demonstrating that AliA and/or AliB are essential for archaeal protein prenylation. Notably, Δ*aliA* showed more severe defects than Δ*aliB* in both lipoprotein biogenesis and cell physiology, and the defect of one could not be complemented by overexpressing the other, indicating different enzymatic activities of the two paralogs. One possible explanation is that AliA serves as the primary enzyme for forming the thioether-linked archaeol, similar to the case of the model bacterium *Mycobacterium smegmatis*, where one Lgt homolog is substantially more active than the other (45, 46). This is further supported by the observation that two conserved arginine residues in AliA correlate with Lgt arginine residues known to be critical for binding to negatively charged membrane lipids (47, 48), as revealed by visual structural comparison (43), whereas these positions are replaced by two histidines in AliB (43). Alternatively, AliA may act on a broader set of substrates than AliB, even if both possess comparable catalytic activity.

Here, we aimed to uncover mechanisms underlying the phenotypic differences between Δ*aliA* and Δ*aliB*. Using proteome-wide Triton X-114 extraction and subsequent mass spectrometry (MS), we systematically assessed the lipidation status of predicted lipoproteins in *Hfx. volcanii* wild-type and *ali* deletion strains. This approach not only resolved the distinct substrate specificities of AliA and AliB, but also substantially expanded the catalog of experimentally validated archaeal lipoproteins. In parallel, lipid-specific MS revealed a marked reduction in thioether-linked archaeol modifications in Δ*aliA*, but not in Δ*aliB*. Together, these findings establish AliA as the dominant contributor to lipoprotein lipidation in *Hfx. volcanii*.

## Results

### Triton X-114 extraction enriched hydrophobic proteins in *Hfx. volcanii*

To determine substrate specificities of AliA and AliB in *Hfx. volcanii*, we set out to examine the lipidation status of lipobox-containing proteins (*i.e.* predicted lipoproteins, hereafter referred to as lipoproteins) at the proteome scale and identify those with impaired lipidation in Δ*aliA*, Δ*aliB*, and Δ*aliA*/Δ*aliB*. Previously, we successfully applied Triton X-114 (TX-114), a detergent widely used in eukaryotes (49, 50, 51) and bacteria (52, 53, 54) to partition hydrophobic proteins into the Triton X-114 (TX) phase and hydrophilic proteins into the aqueous (AQ) phase, to separate the lipidated and non-lipidated forms of two archaeal lipoproteins (43). However, the efficiency of TX-114 for proteome-wide separation of hydrophobic and hydrophilic proteins in archaea has not yet been established.

Therefore, we performed TX-114 extraction in wild-type *Hfx. volcanii* (strain H53) and analyzed the proteome of the two phases *via* label-free, quantitative proteomics. Across the TX and AQ phases, 2724 (64%) of the 4222 predicted proteins encoded by *Hfx. volcanii* were quantified, achieving a high proteome coverage comparable to that of the most comprehensive single dataset in the Archaeal Proteome Project (ArcPP) (see Fig. S1 for a comparison of peptide and protein identifications) (55, 56). This extensive coverage enabled a systematic assessment of protein partitioning between the two phases. As expected, protein groups with different levels of hydrophobicity exhibited marked differences in their distribution. As a positive control, proteins with two or more predicted transmembrane domains (hereafter referred to as ≥2 TM) were highly enriched in the TX phase (median log_2_FC of 4.12, Fig. 1, *A* and *B*). In contrast, the vast majority of predicted cytoplasmic proteins (hereafter referred to as Cyt, defined as proteins lacking predicted transmembrane domains and secretion signals) served as a negative control and were more abundant in the AQ phase (median log_2_FC of −1.27, Fig. 1, *A* and *B*). Consistent with the overall high proteome coverage, 87 (81%) of the 108 lipoproteins predicted by SignalP 6.0 with ≥90% confidence (Data S1) were quantified, with the majority enriched in the TX phase as expected. Specifically, 81 (93%) of the 87 quantified lipoproteins were more abundant in the TX (Fig. 1*A*) than in the AQ phase, including 13 detected exclusively in the TX phase and 68 that were strongly enriched in the TX phase (Table S1). Among lipoproteins showing significant abundance differences between the two phases, the median log_2_FC was 5.59 (Fig. 1*B*), which was significantly higher than that of Cyt proteins (median log_2_FC = −1.27; *p*-value < 10^−10^) and even slightly higher than that of ≥2 TM proteins (median log_2_FC = 4.12; *p*-value < 10^−3^). Together, these results establish TX-114 extraction as an effective method to enrich hydrophobic proteins, providing a foundation for its application in analyzing lipoproteins across strains.

**Figure 1:**
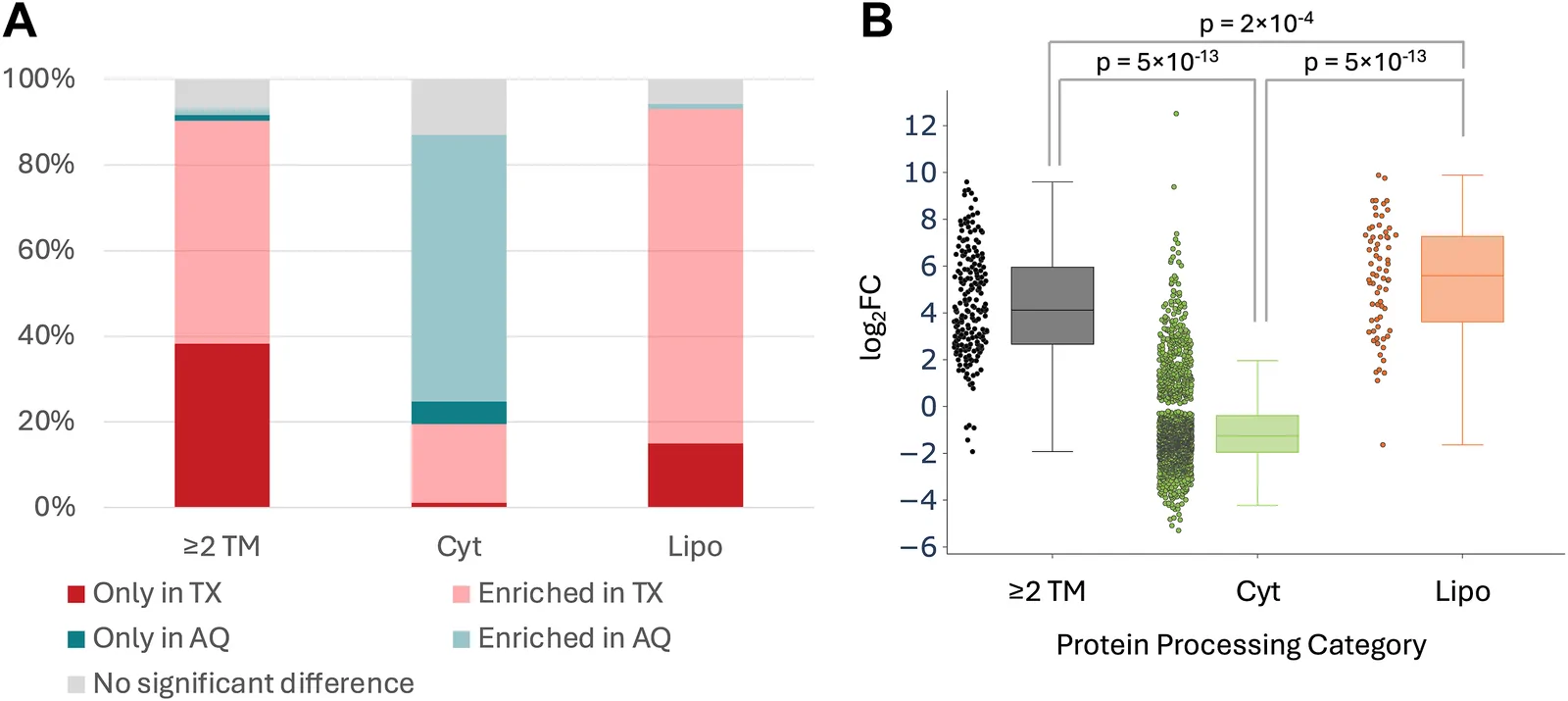
**TX-114 efficiently enriched hydrophobic proteins in wild-type *Hfx. volcanii* (strain H53).***A*, the stacked bar graph indicates the percentage of proteins that were quantified only in the TX-114 (TX) or aqueous (AQ) phase, or enriched in either phase, or showing no significant difference between the phases. Percentages for each predicted protein group are normalized to the total number of quantified proteins within that group. ≥2 TM, proteins with at least two predicted transmembrane domains; Cyt, cytoplasmic proteins; Lipo, lipoproteins. *B*, log_2_ fold-changes (FC) of protein abundance in TX *versus* AQ phase in wild-type *Hfx. volcanii* for different predicted protein groups are depicted as boxplots. Only proteins with significant abundance differences between the two phases are represented in the figure. Boxplots show the median (*center line* between boxes), interquartile range (boxes), as well as the range of data points within 1.5× of the interquartile range (whiskers). Protein abundance differences with *p*-value ≤ 0.05, as determined using a linear mixed model *via* MSstats with subsequent Benjamini-Hochberg correction, were considered significant. Differences between the predicted protein groups were assessed using one-way ANOVA (*p*-value = 5 × 10^−283^) with Tukey’s honestly significant difference test (adjusted *p*-values depicted above brackets).

### *AliA* deletion significantly reduced overall lipoprotein hydrophobicity

We next analyzed the protein profiles in the TX phase and AQ phase of Δ*aliA*, Δ*aliB*, and *ΔaliA*/Δ*aliB* and compared them with the corresponding protein profiles observed in the wild type. A total of 88 lipoproteins were quantified across all four strains (Table S2) Although similar numbers of lipoproteins were quantified in each strain (Fig. 2*A*, Table S2), the fraction that was either enriched or detected exclusively in the TX phase dropped from 92% (81 of 88) in both wild type and Δ*aliB*, to 48% (42 of 88) and 45% (40 of 88) in Δ*aliA*/Δ*aliB* and Δ*aliA*, respectively (Table S2). Accordingly, lipoproteins in the *aliA* deletion strains were increasingly enriched in the AQ phase or showed no significant difference between the two phases (Fig. 2*A*, Table S2). While around 45% of lipoproteins remained associated with the TX phase in *aliA* deletion strains, this distribution does not necessarily indicate lipidation, as non-lipidated lipoproteins may still associate with the TX phase *via* the hydrophobic domain (H-domain) of their uncleaved signal peptides. Indeed, the median log_2_FC of lipoproteins showing significant abundance differences between the two phases decreased from 5.59 in the wild type to 1.37 in Δ*aliA*/Δ*aliB* and 1.39 in Δ*aliA*, reflecting an approximately 16-fold reduction in TX phase enrichment relative to the AQ phase, and thus a significant decrease in lipoprotein hydrophobicity (Fig. 2*B*, *p*-values < 10^−10^ for both strains compared to the wild type). In contrast, Δ*aliB* showed no significant change in log_2_FC (5.59 *versus* 5.95 in wild type, *p*-value = 0.98, Fig. 2*B*), suggesting that AliB has a much narrower substrate range than AliA or may function in processes other than lipidation. It should also be noted that no significant change in the TX/AQ distribution was observed for proteins with ≥2 TM domains in any of the mutant strains, indicating that the reduced protein hydrophobicity in Δ*aliA* and Δ*aliA*/Δ*aliB* is specific to lipoproteins (Fig. 2*B*).

**Figure 2:**
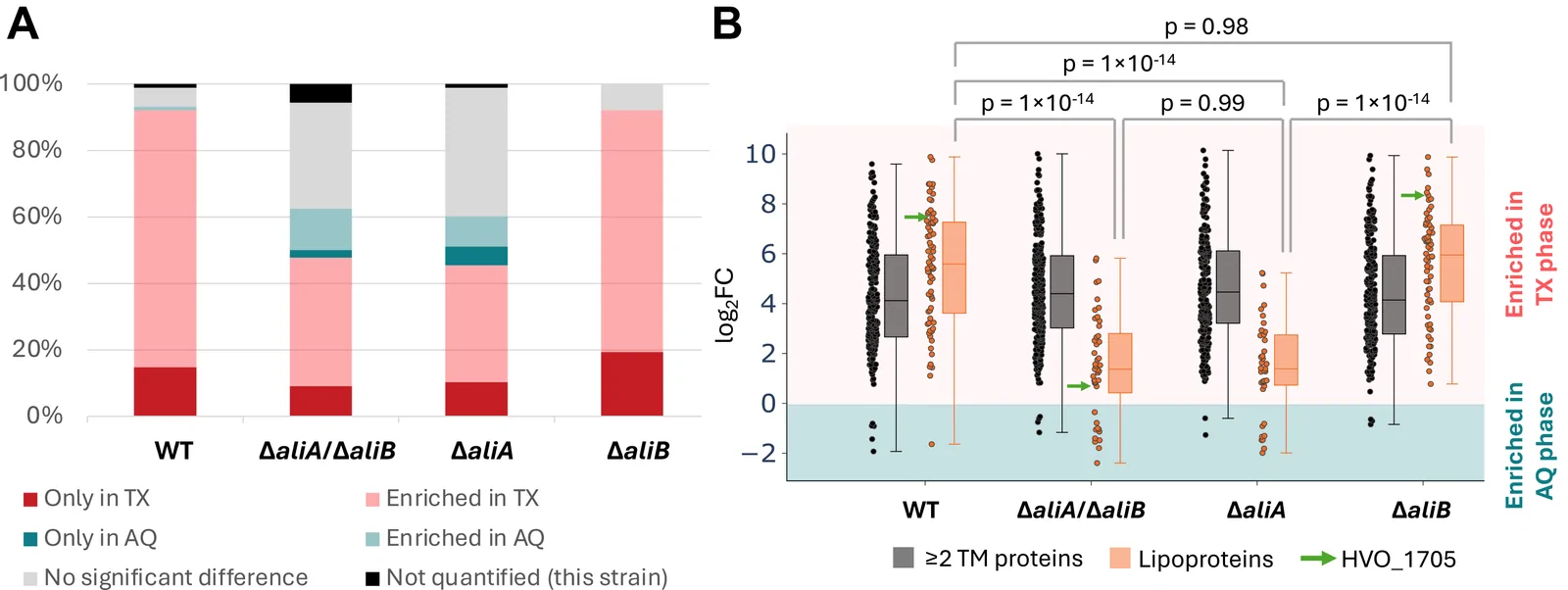
**Deletion of *aliA*, but not *aliB*, substantially reduced the number of TX-enriched lipoproteins.***A*, the stacked bar graph indicates the percentage of lipoproteins quantified only in the TX-114 (TX) or aqueous (AQ) phase, or enriched in either phase, or showing no significant difference between the phases, across different strains. Percentages for each strain are normalized to the total of 88 predicted lipoproteins quantified in at least one of the four strains. “Not quantified (this strain)” corresponds to the percentage of lipoproteins that have been quantified in other strains but not in the respective strain. *B*, log_2_ fold-changes (FC) of protein abundance in TX *versus* AQ phase in the analyzed strains for lipoproteins and ≥2 TM proteins (having at least two predicted transmembrane domains). Only proteins with significant abundance differences are represented in the figure. Protein abundance differences with *p*-value ≤ 0.05, as determined using a linear mixed model *via* MSstats with subsequent Benjamini-Hochberg correction, were considered significant. Differences between the strains were assessed using one-way ANOVA, resulting in a *p*-value of 0.54 for TM proteins and 7 × 10^−28^ for lipoproteins; adjusted *p*-values after Tukey’s honestly significant difference test are shown above the brackets. HVO_1705 is a verified Tat-transported lipoprotein (43). The difference in its abundance between the TX and AQ phases in Δ*aliA* did not reach statistical significance (log_2_FC = 0.35; *p*-value = 0.14) and was therefore not included in panel *B*. Boxplots show the median (*center line* between boxes), interquartile range (boxes), as well as the range of data points within 1.5× of the interquartile range (whiskers).

### Large-scale proteomic validation of the predicted *Hfx. volcanii* lipoproteome

While several studies have predicted the prevalence and abundance of lipoproteins in archaea (4, 41, 43), only a limited number of predicted lipoproteins have been experimentally validated (4, 42, 43, 44). To confirm additional lipoproteins, we applied a three-step validation strategy. First, we selected predicted lipoproteins that, in the wild-type sample, were significantly (*p*-value ≤ 0.05) enriched in the TX phase compared to the AQ phase. From this subset, we identified proteins showing a significant reduction (at least 4-fold) of TX phase abundance in any of the three *ali* mutants compared to the wild type (defined as log_2_FC of TX_mut_:TX_WT_ < −2, *p*-value ≤ 0.05). Third, we excluded proteins that also exhibited reduced abundance in mutant AQ phase, thereby removing those non-lipoproteins with globally decreased expression in *ali* mutants and minimizing false positives. Together, this pattern was interpreted as indicative of reduced protein hydrophobicity in *ali* mutants and as supporting evidence for lipoprotein identity.

To validate this approach, we first assessed how previously characterized predicted lipoproteins performed in this analytical workflow. Four out of six previously confirmed *Hfx. volcanii* lipoproteins were successfully validated using these criteria (Data S2; see Discussion for the two exceptions), supporting the robustness of the method for validating lipoprotein predictions. Besides HVO_1705, HVO_0564, and HVO_1242, this also included HVO_1245, which is predicted as cytoplasmic by SignalP 6.0; however, our MS data support its lipoprotein identity, highlighting the potential of this method for improving lipoprotein prediction tools.

Having validated the workflow, we applied it to all 88 predicted lipoproteins quantified across the dataset. Fifty-four proteins remained after applying the three selection criteria, supporting their predictions as lipoproteins (Fig. 3*A*). The corresponding protein intensity values are provided in Data S3, and heatmaps of z-score-transformed intensities are shown in Fig. S2. Most (49 out of 54) proteins showed reduced hydrophobicity in strains lacking *aliA* (Δ*aliA* and Δ*aliA*/Δ*aliB*), but not in Δ*aliB* (Fig. 3*A*, Table S3), indicating that AliB is involved in the lipidation of substantially fewer *Hfx. volcanii* lipoproteins than AliA. This group includes HVO_1705, a previously characterized lipoprotein that showed impaired lipidation in Δ*aliA* and Δ*aliA*/Δ*aliB* but remained largely unaffected in Δ*aliB* (43) (Fig. 2*B*). In addition, two lipoproteins appeared to be uniquely affected in Δ*aliA* and two in Δ*aliA*/Δ*aliB* (Fig. 3*A*, Table S3). However, these apparent distinctions were driven by missing values (Fig. S2) rather than clear differences in protein abundance and therefore do not provide strong evidence for biological differences between the two mutants. Only one validated lipoprotein was affected by *aliB* deletion. This protein was also affected in Δ*aliA* and Δ*aliA*/Δ*aliB,* consistent with previous results indicating that AliB plays a relatively minor role in *Hfx. volcanii* lipoprotein lipidation (Fig. 3*A*, Table S3).

**Figure 3:**
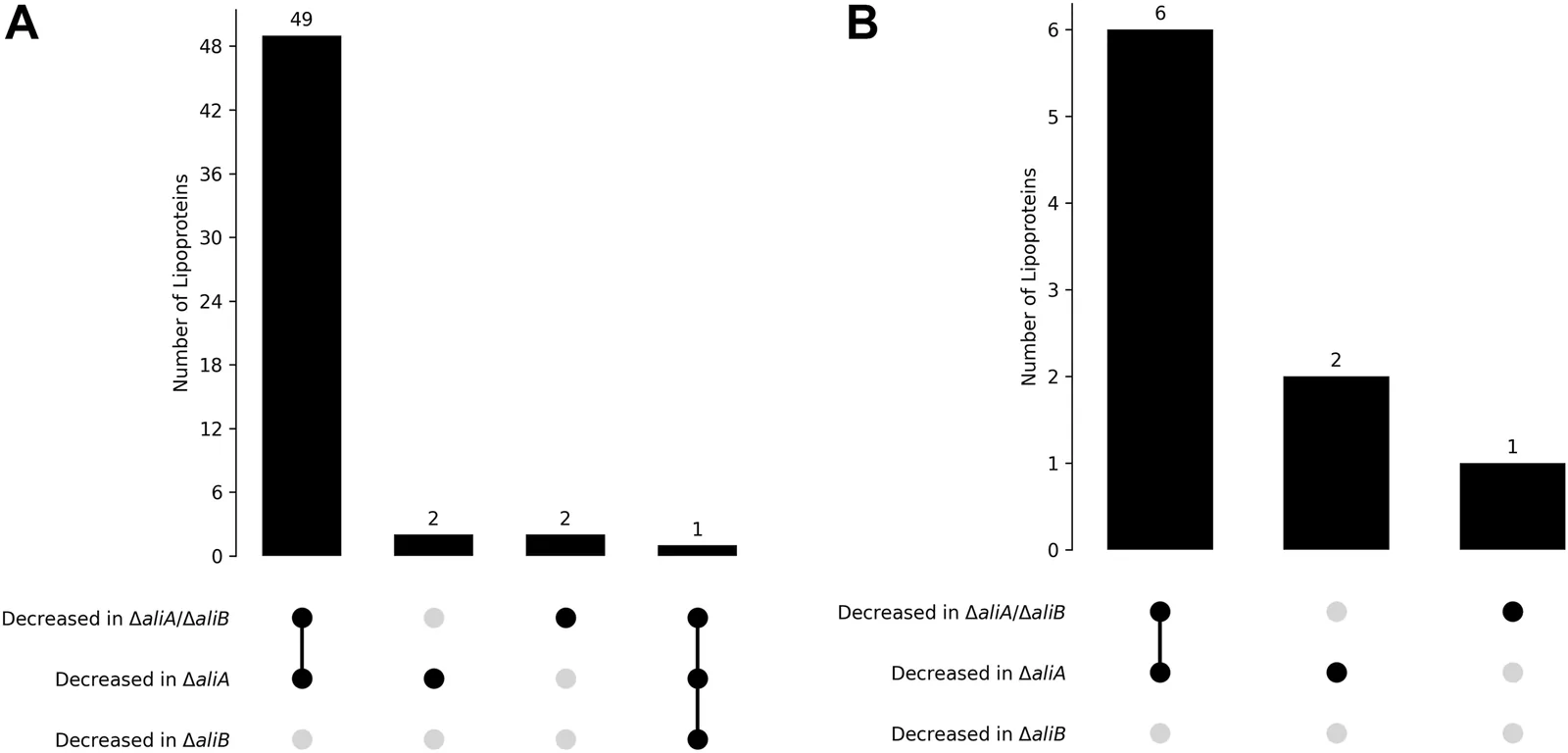
**Large-scale identification of lipoproteins through their reduced TX-phase abundance in *ali* deletion strains.***A*, numbers of lipoproteins that were enriched in wild-type TX phase and exhibited at least a 4-fold reduction in TX phase abundance in Δ*aliA*/Δ*aliB*, Δ*aliA*, or Δ*aliB* compared to the wild type, but were not significantly decreased in the corresponding AQ phase. These proteins are considered validated lipoproteins. *B*, numbers of lipoproteins identified using the extended criteria, including those not quantified in the wild-type AQ phase or the mutant TX phase. Protein abundance differences with *p*-value ≤ 0.05, as determined using a linear mixed model *via* MSstats with subsequent Benjamini-Hochberg correction, were considered significant.

The three-step workflow required statistical comparisons between TX and AQ phases in the wild type and between TX phases of wild-type and mutant strains, and thus proteins had to be quantified in all relevant fractions to meet the significance criteria. Consequently, proteins with missing values in either the AQ phase of the wild type or the TX phase of the mutant could not be evaluated in the initial analysis. However, such missing values are themselves consistent with strong TX-phase enrichment in the wild type or AQ-phase enrichment in the mutant, and therefore consistent with the expected lipoprotein behavior. We therefore applied extended criteria to capture these cases, resulting in the identification of seven additional validated lipoproteins (Fig. 3*B*, Table S3, Data S4, Fig. S3). In addition, two proteins included in Figure 3*B*, HVO_B0034 and HVO_0066, had already been identified in Figure 3*A*. HVO_B0034 was detected in Δ*aliA*/Δ*aliB* in the first analysis but met the extended detection criteria only for Δ*aliA* in this subset, whereas HVO_0066 showed the opposite pattern (Table S3). Among the nine proteins, six exhibited reduced hydrophobicity in both Δ*aliA* and Δ*aliA*/Δ*aliB*, while two were affected only in Δ*aliA*, and one only in Δ*aliA*/Δ*aliB* (Fig. 3*B*, Table S3). Together, these results substantially expand the set of validated *Hfx. volcanii* lipoproteins from fewer than ten to over sixty and further underscore the marked difference in lipoprotein lipidation capacity between AliA and AliB.

### Localization and abundance of the Tat lipoprotein HVO_1705 differ between Δ*aliA* and Δ*aliB*

Building on the global differences observed across the lipoproteome, we next focused on an individual lipoprotein to further explore the distinct roles of AliA and AliB. Current proteomic data indicate that deletion of *aliA*, but not *aliB*, affects the lipidation of the lipoprotein HVO_1705 (Fig. 2*B*, Table S3). This is consistent with previous Western blot analyses, which suggest that deletion of *aliB* has only a minor effect on HVO_1705 lipidation but a more pronounced effect on its signal peptide cleavage (43). These observations highlight HVO_1705 as a suitable model for dissecting AliA- and AliB-dependent steps in lipoprotein biogenesis. Although direct MS analysis of purified HVO_1705 was limited by its low abundance and purity, using the Myc-tagged protein, we observed pronounced differences in its cellular localization and signal-peptide processing among strains. Cell fractionation of wild-type cells overexpressing HVO_1705 showed that the protein was predominantly membrane-associated (Fig. 4*A*). In contrast, in both Δ*aliA*/Δ*aliB* and Δ*aliA* strains, HVO_1705 was predominantly detected in the cytoplasmic fraction as a precursor form with slower electrophoretic mobility and exhibited reduced overall abundance (Fig. 4, *A* and *B*). These observations suggest that *aliA* deletion either impairs membrane targeting of overexpressed HVO_1705 or triggers the clearance of non-lipidated HVO_1705 from both the membrane and cytoplasm *via* an unidentified quality-control mechanism. Conversely, in the Δ*aliB* strain, the majority of HVO_1705 remained membrane-associated, including both the mature form and the presumed lipidated but non-cleaved intermediate form previously reported (43). The intermediate form exhibited slower electrophoretic mobility, likely due to the increased hydrophobicity conferred by lipidation and retention of the signal peptide. This pattern further confirmed that AliB contributes more prominently to signal peptide cleavage than to lipidation of HVO_1705. As a control, we examined the Tat-translocated non-lipoprotein HVO_0844, which is membrane-anchored by a C-terminal transmembrane segment rather than covalent lipid modification. Its membrane localization was unaffected by deletion of *aliA* or *aliB* (Fig. 4, *C* and *D*), confirming that the localization effect observed in the mutants is specific to lipoproteins. Collectively, these results further highlight the distinct roles of AliA and AliB and suggest that *aliA* deletion may additionally reduce lipoprotein abundance through a mechanism that remains to be elucidated.

**Figure 4:**
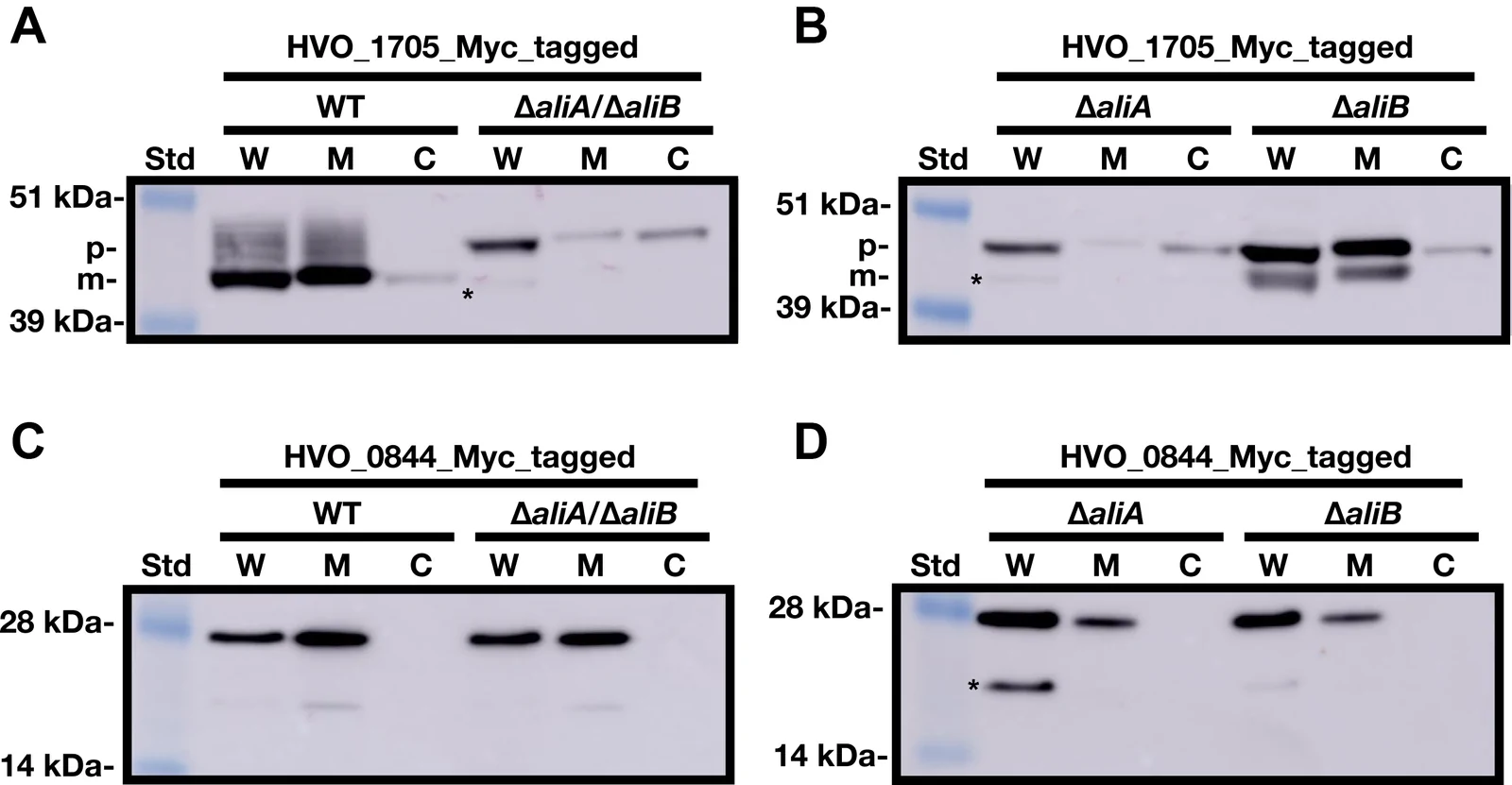
**Deletion of *aliA*, but not *aliB*, led to a reduced amount of membrane-associated HVO_1705, while the Tat-transported transmembrane protein HVO_0844 was unaffected by either deletion.** Western blot probed using an anti-Myc antibody of overexpressed Myc-tagged HVO_1705 in (*A*) wild type, Δ*aliA*/Δ*aliB*, (*B*) Δ*aliA*, and Δ*aliB* in different cell fractions across strains. Western blot of overexpressed Myc-tagged HVO_0844 in (*C*) wild type, Δ*aliA*/Δ*aliB*, (*D*) Δ*aliA*, and Δ*aliB* in different cell fractions across strains. For each target protein, equal amounts of sample were loaded across all lanes, with each lane representing an equivalent proportion of the original cell lysate. Std, SeeBlue Plus2 pre-stained protein standard. W, whole cell lysates. M, membrane fraction. C, cytoplasmic fraction. Precursors (p) and mature proteins (m) are labeled accordingly. The asterisk indicates a likely degradation product of HVO_0844 and HVO_1705, as previously reported for HVO_0844 in our work (43) and for HVO_1705 by Giménez *et al.* (42), respectively. The results are representative of two independent experiments. The original blots and corresponding Ponceau S staining for panels *A-D* are provided in the Source Data file.

### AliA is the main enzyme for thioether-linked archaeol modification of *Hfx. volcanii* lipoproteins

The results presented above focused on dissecting the differences between AliA and AliB using protein-based readouts. To complement these findings, we next examined their roles by directly analyzing lipid modifications across different strains. The lipid attached to the lipobox cysteine *via* a thioether bond can be released by methyl iodide treatment, generating methylthio-archaeol. Previously, we detected methylthio-archaeol in the TX-114 extract of the wild-type strain, whereas this compound was absent in Δ*aliA*/Δ*aliB* (43). To determine whether AliA, AliB, or both were responsible for this phenotype, we performed liquid chromatography-mass spectrometry (LC-MS) analysis of lipids isolated from TX-114 extracts of Δ*aliA* and Δ*aliB* strains. Deletion of *aliA* alone resulted in the complete absence of methylthio-archaeol, while deletion of *aliB* did not reduce the level of thioether-linked archaeol modification (Fig. 5, *A* and *B*). This result correlates well with our observation that deletion of *aliA* reduced the hydrophobicity of substantially more *Hfx. volcanii* lipoproteins than deletion of *aliB* (Fig. 3). Thus, the loss of this lipid modification in the Δ*aliA*/Δ*aliB* strain (43) is primarily attributable to *aliA* deletion, underscoring the essential role of AliA in mediating this lipid modification (Fig. 5, *A* and *B*). As a control, we analyzed the total lipid composition of each strain. Comparable archaeol profiles were observed across all strains, including the wild type (Fig. 5, *C* and *D*), indicating that the absence of methylthio-archaeol in *aliA* deletion strains was not due to impaired archaeol synthesis but instead reflects a defect in the formation of the thioether-linked archaeol modification.

**Figure 5:**
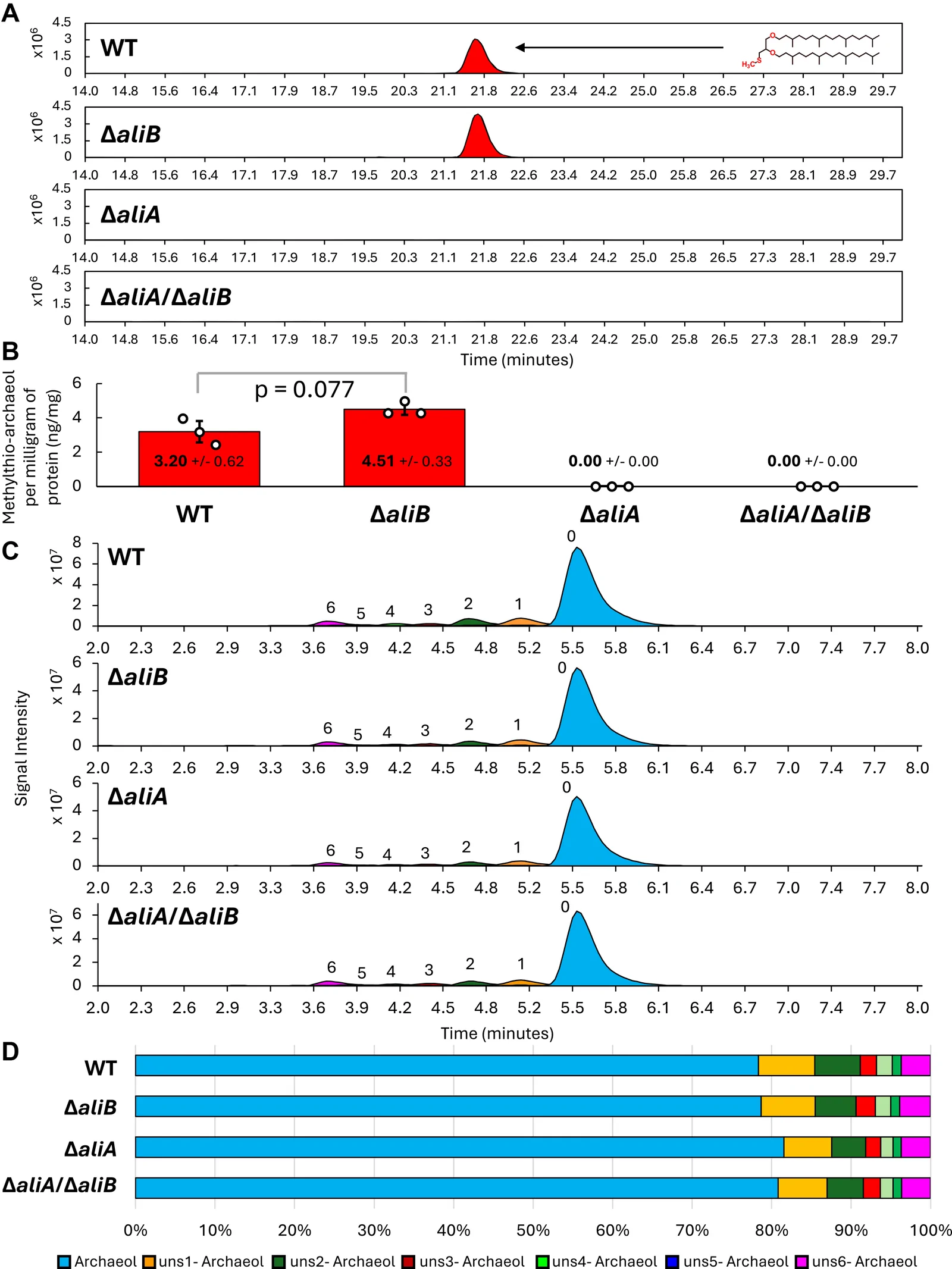
**Deletion of *aliA*, rather than *aliB*, led to the complete absence of thioether-linked archaeol in lipoprotein extracts.***A*, representative merged extracted ion chromatograms (EICs, m/z values in Experimental procedures) showing that the methylthio-archaeol is readily detectable in extracts from methyl iodide treated lipoproteins from the wild type and Δ*aliB*, while methylthio-archaeol is notably absent in that from the Δ*aliA* and Δ*aliA*/Δ*aliB* strains. *B*, bar chart showing the average mass of methylthio-archaeol recovered from methyl iodide treated lipoproteins per milligram of starting protein, based on three biological replicates. Error bars represent standard deviations. Statistical significance was assessed using a two-tailed unpaired *t* test assuming unequal variances (Welch’s *t* test) for comparison between the wild type and Δ*aliB* (*p* = 0.077). *C*, as a control, the total lipids of each strain were analyzed. Representative merged EICs (m/z values in Experimental procedures) showed the same saturated and unsaturated diether core lipids (archaeols) in base-hydrolyzed biomass from all strains. *D*, average (n = 3) relative abundance of saturated and unsaturated diether core lipids (archaeols) in base hydrolyzed biomass were comparable across all strains.

### Downstream effects of *aliA* and *aliB* deletion beyond lipoproteins

Finally, we examined the effects of *aliA* and *aliB* deletion on non-lipoproteins, as changes in their abundance may provide insight into the molecular basis of the phenotypes observed in Δ*ali* mutants (43). Moreover, differentially abundant non-lipoproteins may represent previously unrecognized components of the archaeal lipoprotein biogenesis pathway.

To assess the global impact of *ali* deletion on the *Hfx. volcanii* proteome, we first performed principal component analysis (PCA) and Pearson correlation analysis (Fig. S5). Both analyses revealed high proteomic similarity among the AQ fractions of the different strains but pronounced differences among the TX fractions. Consistent with the central role of AliA in lipoprotein lipidation, Δ*aliA* and Δ*aliA*/Δ*aliB* clustered closely together and separately from WT and Δ*aliB*, although Δ*aliB* still exhibited noticeable proteome-wide differences relative to WT. We next identified non-lipoproteins enriched in either the TX or AQ phase that exhibited significant abundance changes upon *ali* deletion. Differentially abundant proteins exhibiting significant finite log_2_FC in one phase relative to the other (*i.e.*, detected in both phases but enriched in one) are shown in the top panels of Figure 6 (TX phase) and Figure 7 (AQ phase). Proteins detected exclusively in the one phase—resulting in infinite log_2_FC due to missing data in the other—are shown in the bottom panels of Figure 6 and Figure 7. These latter comparisons rely on presence/absence behavior and missing values and are therefore less quantitatively robust than the finite data. Nevertheless, they highlight strongly phase-specific proteins.

**Figure 6:**
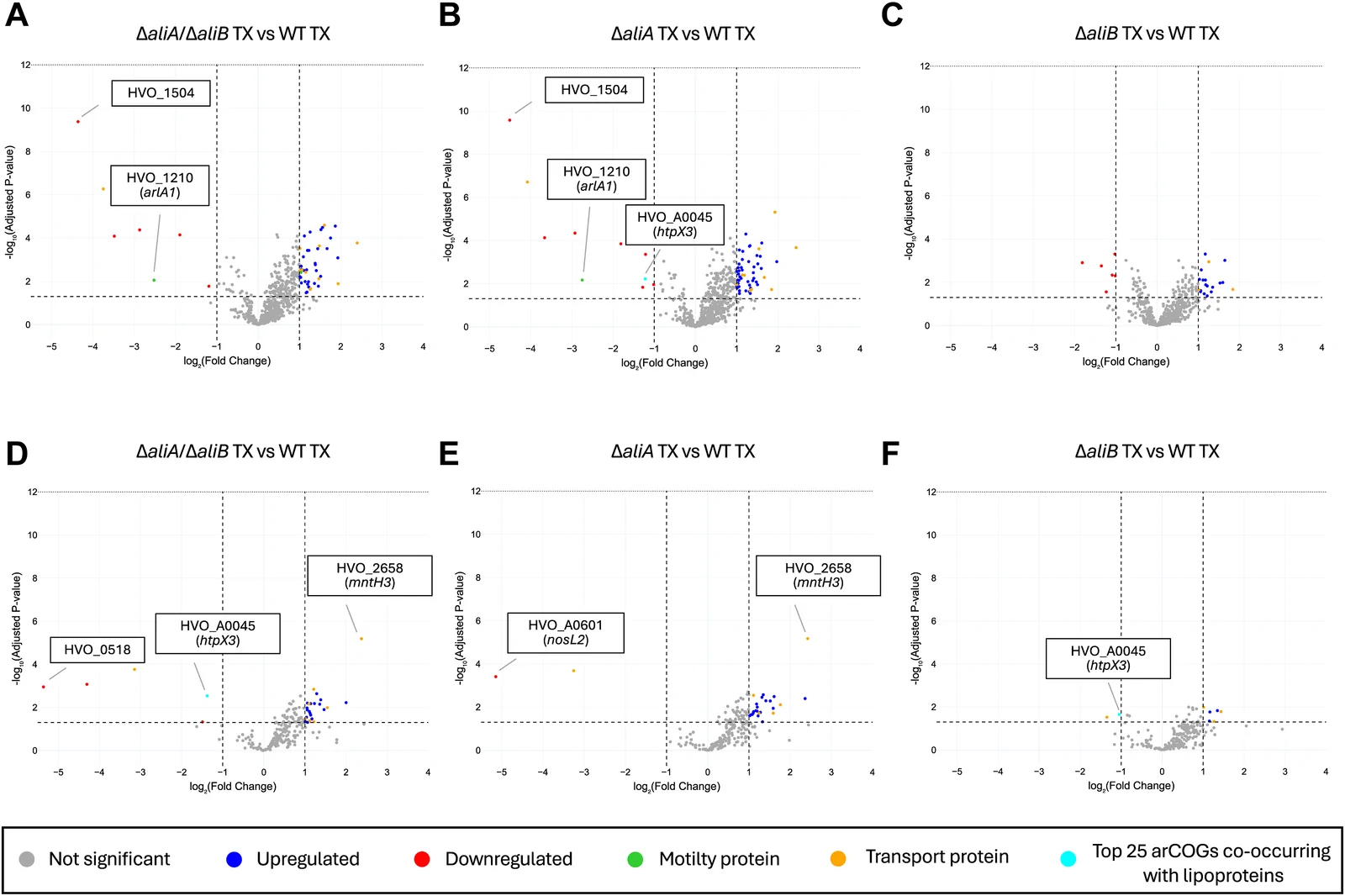
**Deletion of *aliA* and *aliB* predominantly perturbs TX-associated non-lipoproteins.** Volcano plots show proteins in the TX phase of (*A*, *D*) Δ*aliA*/Δ*aliB*, (*B*, *E*) Δ*aliA*, and (*C*, *F*) Δ*aliB* compared to wild-type *Hfx. volcanii*. Panels *A–C* display proteins exhibiting significant finite log_2_FC in TX relative to AQ within each strain (*i.e.*, detected in both fractions but enriched in TX). Panels *D–F* display proteins detected exclusively in the TX phase of at least one strain, resulting in infinite log_2_FC due to absence from the AQ phase. Proteins with |log_2_FC| > 1 and adjusted *p*-value < 0.05, as determined using a linear mixed model *via* MSstats with subsequent Benjamini-Hochberg correction, are considered significantly differentially abundant. Selected groups of differentially abundant proteins are highlighted in distinct colors as indicated.

**Figure 7:**
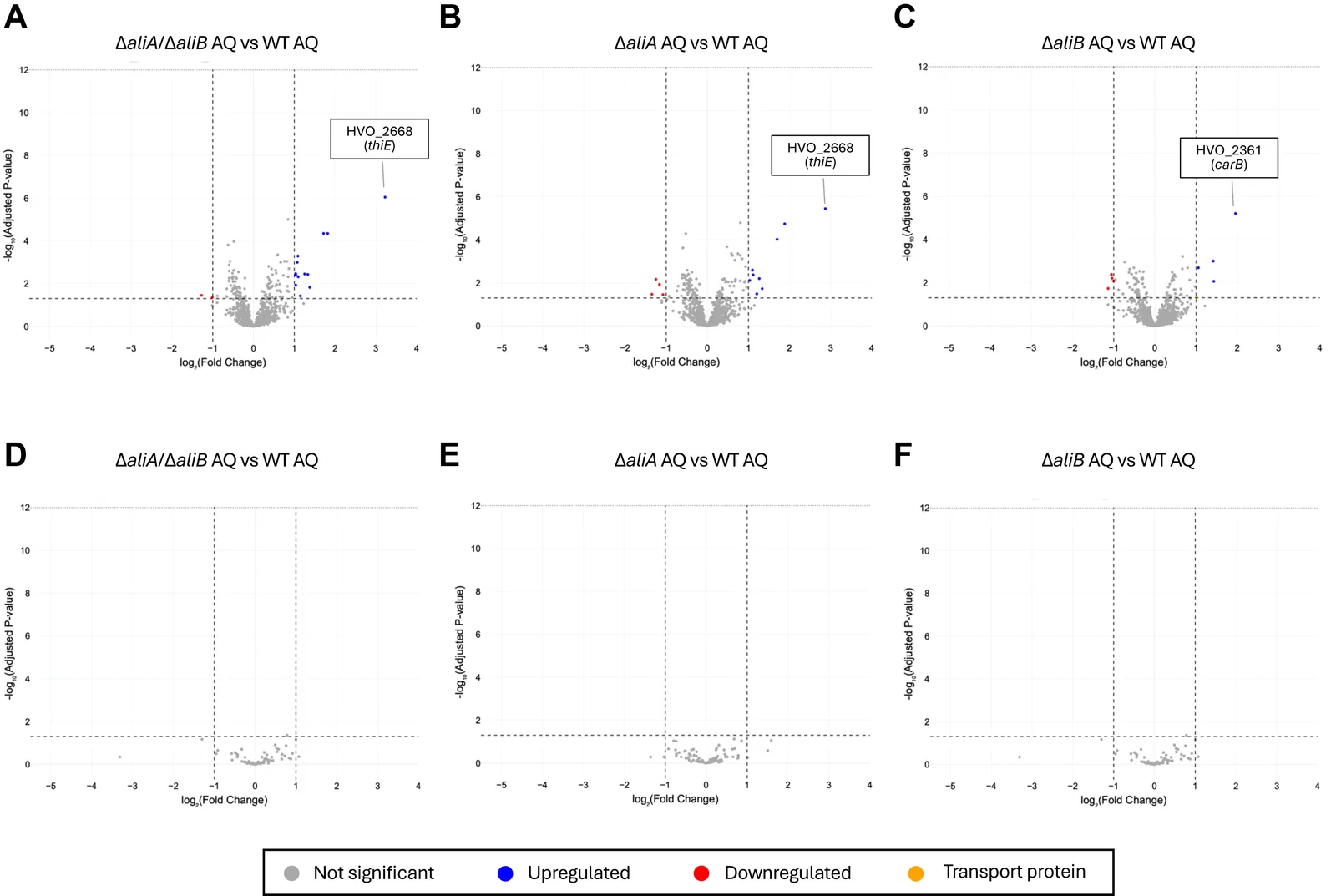
**Deletion of *aliA* and *aliB* induces comparatively modest changes in AQ-associated non-lipoproteins.** Volcano plots show proteins in the AQ phase of (*A*, *D*) Δ*aliA*/Δ*aliB*, (*B*, *E*) Δ*aliA*, and (*C*, *F*) Δ*aliB* compared to wild-type *Hfx. volcanii*. Panels *A–C* display AQ-associated proteins exhibiting significant finite log_2_FC in AQ relative to TX within each strain (*i.e.*, detected in both phases). Panels *D–F* display proteins detected exclusively in the AQ phase of at least one strain, resulting in negative infinite log_2_FC due to absence from the TX phase. Proteins with |log_2_FC| > 1 and adjusted *p*-value < 0.05, as determined using a linear mixed model *via* MSstats with subsequent Benjamini-Hochberg correction, are considered significantly differentially abundant. No significantly differentially abundant proteins were detected in panels *D-F*. Selected functional groups of differentially abundant proteins are highlighted in distinct colors as indicated.

Consistent with the PCA and the Pearson correlation analysis, the most pronounced differences were observed in the TX phase, which contained both a great number of differentially abundant proteins and a wide range of log_2_FC (Fig. 6, Data S5–S10; see Data S12 for interactive volcano plots). In contrast, the AQ phase displayed comparatively fewer and generally more moderate changes (Fig. 7, Data S11). This pattern indicates that disruption of Ali-dependent lipidation primarily perturbs the membrane-associated proteome rather than the soluble protein pool. Among the TX-associated proteins, the archaellin subunit protein ArlA1 (HVO_1210) remained enriched in the TX phase of *aliA* deletion strains, but its abundance in the TX phase was reduced compared to the wild type (Fig. 6, *A* and *B*), providing a molecular explanation for their previously reported motility defects (43). In addition, numerous transport proteins were more abundant in the TX phase of the mutant strains than in the wild type (Fig. 6). As many substrate-binding proteins of the ABC transport systems are predicted lipoproteins (Data S1), the increased abundance of transport proteins may represent a compensatory response to impaired lipidation and function of these substrate-binding proteins.

Previously, we identified a comprehensive set of arCOGs (Archaeal Clusters of Orthologous Genes) (57, 58) that most frequently co-occur with lipoproteins as promising candidates for archaeal lipoprotein biogenesis components, including arCOG02177 (*aliA*) (43). We therefore assessed whether the top 25 arCOGs from the list showed different abundance between mutant and wild-type strains. Among these, HVO_A0045 (arCOG01331), encoding an HtpX-like protease, showed reduced abundance in Δ*aliA* when taking only finite log_2_FC into account (Fig. 6*B*). When proteins that were only detected in the TX phase were also considered, decreased abundance of HVO_A0045 was additionally observed in Δ*aliA*/Δ*aliB* and Δ*aliB* strains (Fig. 6, *D* and *F*). Bacterial HtpX homologs are implicated in the degradation of misfolded envelope proteins and undergo self-cleavage upon membrane perturbation (59), suggesting that altered abundance of this protease may reflect changes in envelope protein homeostasis following disruption of Ali-dependent lipidation. Whether the altered abundance of the protein reflects direct consequences of Ali pathway disruption, indirect effects stemming from impaired lipoprotein maturation and associated cellular stress, or loss of function of individual lipoproteins remains to be resolved. Finally, we analyzed the abundance of the nine known ArtA substrates and ArtA itself in the mutants (31, 60). While ArtA and seven of nine substrates were detected in our datasets, none showed significant abundance differences (Table S4), suggesting that the ArtA-dependent prenylation pathway operates largely independent of the AliA/AliB-dependent lipoprotein lipidation pathway in *Hfx. volcanii*.

## Discussion

Protein lipidation occurs across all domains of life, yet the underlying mechanisms in archaea remain poorly understood. Recently, we identified AliA and AliB as the first archaeal enzymes involved in lipoprotein biogenesis and showed their involvement in archaeal lipoprotein lipidation (43). Although the two proteins are paralogs, their functions are not redundant. Deletion of *aliA* or *aliB* in *Hfx. volcanii* led to distinct phenotypes in both lipoprotein biogenesis and cell physiology, and cross-complementation did not restore the defects, indicating that the two enzymes possess different enzymatic activities.

In this study, we further explored the functional differences between AliA and AliB by analyzing the lipidation status of predicted lipoproteins in Δ*aliA*, Δ*aliB*, and Δ*aliA*/Δ*aliB*. We employed a proteome-wide Triton X-114 extraction, which enabled efficient separation of hydrophobic (TX phase,) and hydrophilic (AQ phase) proteins from large culture samples. Subsequent MS of both fractions yielded high proteome coverage, comparable to the most comprehensive single dataset in the Archaeal Proteome Project (Fig. S1, *A* and *B*), underscoring TX-114 extraction as a powerful method for archaeal proteomic analysis, particularly for enhancing the identification of membrane proteins. Overall, TX-114 extraction resulted in the expected separation of hydrophobic and hydrophilic proteins (Fig. 1, *A* and *B*), validating its use for subsequent proteomic analysis on *Hfx. volcanii* lipoproteome. At the same time, a subset of predicted lipoproteins and transmembrane proteins showed enrichment in the AQ phase, while some predicted cytoplasmic proteins were detected in the TX phase. Such fractionation patterns likely reflect a combination of imperfect predictions, minor cross-contamination, protein–protein interactions influencing phase partitioning, or lower-than-expected hydrophobicity of specific lipid modifications or signal peptide H-domains.

This approach also enabled the large-scale validation of archaeal lipoprotein predictions for the first time. In total, 61 of 88 predicted lipoproteins quantified across all strains showed reduced abundance in the TX phase—but not in the AQ phase—in at least one mutant (Fig. 3), verifying their identity as lipoproteins. Three previously experimentally validated lipoproteins were included among the 61 proteins (Data S2); a fourth (HVO_1245) was also supported by the MS data (Data S2) but was not included among the 61 because SignalP 6.0 did not predict it as a lipoprotein. HVO_1808, a predicted Sec lipoprotein, represents another interesting case (Data S2). Previous Western blot analyses of this protein yielded ambiguous results: although its lipobox Cys-to-Ser mutant showed reduced stability, the wild-type protein, Cys-to-Ser mutant, and signal peptide-deleted (Δss) mutant all displayed similar electrophoretic mobilities (4), providing no clear evidence that HVO_1808 undergoes lipidation and is a *bona fide* lipoprotein. Consistent with these observations, our MS data showed only a modest reduction in the TX-phase abundance of HVO_1808 in *aliA* mutants that did not meet our validation threshold, suggesting that its SignalP 6.0 lipoprotein prediction may represent a false positive. Two previously experimentally validated lipoproteins (43), HVO_B0139 and HVO_1176, were not validated by the MS-based workflow (Data S2). HVO_B0139 exhibited significant reduction in TX abundance in all mutants. However, it did not meet the required fold-change threshold and is therefore not considered validated in our analysis. This indicates that the relatively stringent criteria used in our study may exclude some *bona fide* lipoproteins, but they were chosen to minimize potential false-positive assignments, as illustrated by HVO_1808. HVO_1176 did not meet the validation criteria despite previous Western blot analyses indicating impaired maturation and increased AQ-phase enrichment in *ali* deletion strains (43). This discrepancy may reflect differences in the protein forms detected by the two approaches: previous Western blot analyses detected tagged, plasmid-expressed HVO_1176, whereas the present MS analysis quantified peptides derived from endogenous HVO_1176. Thus, overexpression, tagging, or differences in the specific protein forms detected may have contributed to the distinct TX/AQ partitioning patterns observed between the two analyses.

Considering that the current archaeal lipoprotein prediction program is trained on a very limited number of experimentally verified lipoproteins (61), the extensive lipoprotein validation presented here provides a critical dataset for refining and expanding the predictive model. It should be noted, however, that lipoproteins for which failed lipid attachment leads to protein instability are not captured by our analysis, as the applied filtering criteria exclude proteins with strongly reduced overall abundance. Future studies that directly identify lipid-modified peptides by mass spectrometry will further strengthen lipoprotein validation. Consistent with our previous findings that Δ*aliA* exhibited more severe phenotypes in growth, morphology, and motility than Δ*aliB* (43), deletion of *aliA* affected substantially more lipoproteins than deletion of *aliB* (Fig. 3). Specifically, 60 of 61 validated lipoproteins showed reduced TX phase abundance in Δ*aliA*, whereas only one lipoprotein was affected in Δ*aliB*, confirming the dominant role of AliA in *Hfx. volcanii* lipoprotein lipidation (Fig. 3, Table S3).

Complementing the whole-proteome analysis, we examined a Tat-transported lipoprotein HVO_1705 both as proof of concept and as a model substrate to further dissect the roles of AliA and AliB. In Δ*aliA* and Δ*aliA*/Δ*aliB* strains, HVO_1705 abundance was strongly reduced and the protein localized predominantly to the cytoplasmic fraction (Fig. 4, *A* and *B*), suggesting impaired translocation or clearance of the non-lipidated form from the membrane by an unidentified quality-control system. In contrast, HVO_1705 expressed in Δ*aliB* remained largely membrane-associated, including both the mature form and a presumed lipidated but non-cleaved intermediate (Fig. 4*B*). These observations indicate that AliB is not essential for membrane localization and lipidation of HVO_1705 and may instead be involved in signal peptide cleavage of lipoproteins. Elucidating the precise role of AliB in lipoprotein biogenesis will therefore represent an important direction for future studies. Analysis showed that a Tat-transported transmembrane protein, HVO_0844, was unaffected by the deletion of either *ali* gene (Fig. 4, *C* and *D*), confirming that these effects are specific to lipoproteins.

Consistent with the functional distinction between AliA and AliB, LC-MS analysis of lipids isolated from TX-114 extracts further confirmed the dominant role of AliA in mediating thioether-linked archaeol modification in *Hfx. volcanii*. The cleavage product of this modification (methylthio-archaeol) was absent in both Δ*aliA* and Δ*aliA*/Δ*aliB* (Fig. 5, *A* and *B*). Unexpectedly, deletion of *aliB* did not significantly alter the level of thioether-linked archaeol modification compared to the wild type (*p*-value = 0.077, Fig. 5*A*), and the mean methylthio-archaeol level was even slightly increased. We hypothesized this trend may be associated with increased AliA abundance in the absence of AliB. To test this, we performed quantitative PCR, which indicated a non-significant trend toward increased AliA levels in Δ*aliB* (Fig. S4, *p*-value = 0.174), suggesting potential functional crosstalk between AliA and AliB that warrants further investigation.

Beyond the altered abundance of *aliA* in the Δ*aliB* mutant, we observed differential abundance of multiple non-lipoproteins in *ali* deletion strains (Figs. 6 and 7). A considerable number of non-lipoproteins exemplifies differential abundance in *ali* deletion strains, especially in the TX phase (Fig. 6). Among these was HVO_A0045 (Fig. 6), which belongs to an arCOG previously identified as one of those most frequently co-occurring with archaeal lipoproteins (43), although how its respective function relates to the lipoprotein biogenesis pathway remains unclear. As an HtpX-like protease, the reduced abundance of HVO_A0045 in the mutants may reflect its involvement in downstream processes of the Ali pathway, such as releasing membrane-associated lipoproteins to the extracellular environment or activation of its self-cleavage activity in response to membrane stress. Although membrane instability has not been reported for *ali* deletion strains, increased outer membrane permeability has been observed in an *Escherichia coli* Δ*lgt* strain (62). Additional proteins showing differential abundance in the mutants likely reflect broader consequences of Ali pathway disruption beyond lipoproteins themselves, including downstream effects of impaired lipoprotein lipidation. Among these, a large number of transport proteins were affected (Figs. 6 and 7). In addition, the archaellin subunit protein HVO_1210 exhibited reduced abundance in both Δ*aliA* and Δ*aliA*/Δ*aliB* (Fig. 6, *A* and *B*), providing a molecular explanation for the motility defects observed in these strains (43).

In summary, by combining quantitative proteomics with LC-MS analysis of thioether-linked archaeol modifications in wild-type and *ali* deletion strains, we demonstrate that the vast majority of archaeal lipoprotein lipidation in *Hfx. volcanii* is mediated by AliA, with AliB acting in a partially distinct role, likely downstream of lipidation and potentially linked to signal peptide cleavage. Beyond defining the dominant contribution of AliA, our quantitative proteomic analysis revealed changes in the abundance of multiple non-lipoproteins in *ali* mutants, which may point to candidate components or regulators of the archaeal lipoprotein biogenesis pathway. Importantly, this work substantially expands the number of experimentally validated archaeal lipoproteins from fewer than ten to over sixty, providing a robust experimental framework for refining archaeal lipoprotein prediction algorithms. Together, these data offer a valuable community resource for studying protein lipidation across archaea, where the underlying mechanisms have remained largely elusive.

## Experimental procedures

### Growth conditions

Plasmids and strains used in this study are listed in Table S5. *Hfx. volcanii* strains were grown at 45 °C in liquid (orbital shaker at 250 rpm with an orbital diameter of 2.54 cm) or on solid agar (containing 1.5% [w/v] agar) semi-defined Hv-Cab medium (63), supplemented with tryptophan (Fisher Scientific) and uracil (Sigma) at a final concentration of 50 μg ml^−1^ unless otherwise noted. Uracil was left out for strains carrying pTA963-based plasmids.

### Triton X-114 extraction

A single colony of *Hfx. volcanii* was inoculated into 10 ml of liquid medium supplemented with 200 μg ml^−1^ tryptophan. When the culture reached an OD_600_ of 0.3 to 0.8, it was used to inoculate 1 l of secondary culture (starting OD_600_ = 0.004) in the same medium. Cells were harvested at OD_600_ = 0.75 to 0.85 by centrifugation at 7000 × *g* for 20 min at 4 °C. The cell pellets were resuspended in 7 ml PBS (pH 7.4) supplemented with 150 μl RQ1 RNase-Free DNase and protease inhibitor, resulting in a final volume of approximately 10 ml. The cell suspensions were transferred to 15 ml Falcon tubes, vortexed thoroughly, and lysed by five freeze-thaw cycles, alternating between freezing in liquid nitrogen for 2 min and thawing at 37 °C for 20 min. Unlysed cells were removed by centrifugation at 9000 × *g* for 20 min at 4 °C. Protein concentrations of the clarified lysates were determined using the Pierce BCA Protein Assay Kit (Thermo Scientific). Triton X-114 extraction was performed as previously described with slight modifications (43). Briefly, 1 ml of ice-cold 20% Triton X-114 was added to 9 ml of clarified lysate containing 80 mg of total protein in a 15 ml Falcon tube. The mixture was gently mixed on a rotator at 4 °C for 3 h and then centrifuged at 9000 × *g* for 10 min at 4 °C to remove precipitates. The supernatant was transferred to a fresh tube and incubated at 37 °C for 20 min to induce detergent phase separation, followed by centrifugation at 9000 × *g* for 10 min at room temperature. The resulting phase separation yielded approximately 9 ml of aqueous (AQ) phase and ∼1 ml of detergent (TX) phase. The AQ phase was transferred to a new 15 ml Falcon tube, and 889 ul of ice-cold 20% TX-114 was added for washing. The TX phase was adjusted to a final volume of 10 ml with PBS for washing. Both samples were incubated on ice for 10 min until clear and then at 37 °C for 20 min until the solution became turbid. Phase-separated samples were centrifuged again at 9000 × *g* for 10 min at room temperature. The washed AQ phase was transferred to a new tube and stored at −80 °C for further use. For the washed TX phase, the supernatant was removed, 10 ml methanol was added, and samples were stored at −80 °C overnight to precipitate proteins. Protein precipitates were collected the following day by centrifugation at 10,000 × *g* for 10 min and the pellet was stored at −80 °C for downstream analysis.

### Mass spectrometry of proteins in TX and AQ phases

Triton X-114 extraction was performed as described above, starting with cell lysates containing 60 mg of total proteins. Protein precipitates from the TX phase were resuspended with 200 μl or 300 μl PBS buffer with protease inhibitor. The protein concentration was measured using the BCA Protein Assay Kit. Mass spectrometry sample preparation was performed as previously described (64). Briefly, 50 μg of protein from each sample was reduced with DTT, alkylated with iodoacetamide, and digested with trypsin overnight at 37 °C using S-trap mini spin columns (ProtiFi) following manufacturer’s instructions. Resulting peptides were eluted, dried in a vacuum centrifuge, and desalted using C18 stage-tips as described previously (65). Mass spectrometric analysis was performed, as described in Schiller *et al.* (2024) (64), on an Orbitrap Eclipse after separation on a nanoEase M/Z Peptide BEH C18 column. The corresponding chromatography and mass spectrometry settings are listed in Table S6. TX and AQ phase samples from three biological replicates were analyzed.

### Proteomics data processing and statistical analysis

For all proteome-focused analyses described in this study, the theoretical proteome of *Hfx. volcanii* from March 28th, 2023 (https://doi.org/10.5281/zenodo.7794769) was used, comprising 4222 proteins. This theoretical proteome, which reflects a Gold Standard Protein-based genome annotation (66), has been subjected to an extensive analysis to resolve missing gene calls and was initially maintained in HaloLex (67, 68).

Peptide identification and quantification with statistical post-processing followed the same procedures as in Chatterjee *et al.* (2025) (69), except that the above-described newer version of the theoretical proteome was used. Briefly, within Ursgal (version 0.6.9) (70), MS raw files were converted into mzML using the ThermoRawFileParser (version 1.1.2) (71), before conversion to MGF with pymzML (version 2.5.0) (72). Peptides were identified through protein database searches using MSFragger (version 3.0) (73), X!Tandem (version Alanine) (74), and MS-GF+ (version v2019.07.03) (75), and statistical post-processing was performed *via* Percolator (version 3.4.0) (76). After filtering results for a combined PEP ≤1%, peptides were quantified through FlashLFQ (version 1.1.1) (77). Peptide abundances were further processed through MSstats (version 4.0) (78), including log transformation, median normalization, imputation of missing values, and integration on the protein level using the *dataProcess* function. MSstats performed imputation using an accelerated failure time model, which assumes that values are missing due to the low abundance of the corresponding peptides. This approach allows for imputation to be applied for a protein in a mass spectrometry run when at least one measured feature (defined as the combination of peptide sequence and precursor charge) is present for that protein and run. Imputed values were not generated for proteins that are completely absent from a run (Fig. S6); these proteins were considered “Not quantified” in the corresponding phase and strain. Furthermore, fold-changes (FCs) were calculated using the MSstats *groupComparison* function, which employs a linear mixed model to determine *p*-values that were additionally adjusted for multiple testing (using the Benjamini-Hochberg approach). For proteins that were completely absent in one condition, log_2_FCs were given as Inf (protein missing in the denominator condition) or -Inf (protein missing in the numerator condition), including proteins for which no features could be imputed. Log_2_FCs were considered statistically significant if their associated adjusted *p*-value was ≤0.05. Search and quantification parameters are listed in Table S7.

To compare the distribution of different protein groups in the TX and AQ phases, the whole theoretical proteome was used as an input for the prediction of lipoproteins (comprising lipoproteins processed through the Sec as well as the Tat pathway) through SignalP (v. 6.0) (61), transmembrane domains through DeepTMHMM (v. 1.0.44) (79), and type III signal peptides through FlaFind (v. 1.2) (80). Proteins were assigned to the predicted lipoprotein group, if SignalP results for LIPO(Sec/SPII) or TATLIPO(Tat/SPII) had a confidence of ≥0.9, and if they were not FlaFind positive. The ≥2 TM protein group was defined by DeepTMHMM results that predicted at least 2 TM regions, as long as these proteins were not FlaFind positive or exhibited SignalP predictions different from "Other”. All proteins that were FlaFind negative and had “Other” as a SignalP prediction, which in combination means that they do not contain any of the secretion signals and had no predicted TM regions, were designated as predicted cytoplasmic proteins.

MSstats results were grouped based on these predicted protein groups, and figures were generated using custom Python scripts (https://doi.org/10.5281/zenodo.18783633), employing the following packages: Pandas (81), SciPy (82), Plotly (83), and Matplotlib (84). Comparisons between the groups were assessed for statistical significance employing a one-way ANOVA test with Tukey’s *post hoc* test, using the SciPy stats package. Protein groups containing multiple proteins sharing identical peptides were further sanitized using proteotypic peptides, *i.e.* peptides specific to a single protein. If all proteins within a protein group were independently quantified through proteotypic peptides, the corresponding protein group entry was excluded from downstream MSstats analyses.

### Lipid processing and analysis

A single colony of *Hfx. volcanii* was inoculated into 5 ml of liquid medium and grown to an OD_600_ of 0.3 to 0.8. This culture was then used to inoculate 25 ml of fresh medium at a starting OD_600_ = 0.01. Cells were harvested at an OD_600_ = 0.65 to 0.75 by centrifugation at 7000 × *g* for 20 min at 4 °C. Core lipids were obtained from the harvested biomass by base hydrolysis as previously described (43). In brief, cell pellets were hydrolyzed in 1M KOH (dissolved in methanol) for 3 h at 75 °C and then neutralized with HCl. Core lipids were extracted from the hydrolyzed biomass with dichloromethane (DCM). DCM extracts were evaporated under an N_2_ stream and then resuspended in 1 ml of 9:1 methanol:DCM and filtered through 0.45 μm polytetrafluoroethylene (PTFE) filters for analysis *via* reverse-phase (RP) liquid chromatography-mass spectrometry (LC-MS).

For the methyl iodide treatment, lipoproteins were extracted from 80 mg total proteins using Triton X-114. Following extraction, lipoprotein samples were treated with methyl iodide to release thioether bound lipids as previously described (43). To summarize, Triton X-114 extracted proteins were first resuspended in 400 μl of 50 mM Tris-HCl buffer (pH 8.0) and treated with 200 μl 3% formic acid and 600 μl methyl iodide to methylate the sulfur atom mediating the thioether linkage between the lipid and protein (19). Samples were incubated in the dark and shaken at 200 rpm at 37 °C for 24 h. Next, the methyl iodide was evaporated off under N_2_ stream and samples were brought to a final volume of 1 ml by adding ∼400 μl of 50 mM Tris-HCl buffer (pH 8.0). To break the linkage between the protein and methylthio-archaeol (19), the pH of the solution was increased to 12 with 10N NaOH and the incubation was continued for 24 h as before. Following this, the released lipids were extracted with a mixture of 9:1 chloroform:MeOH which was then dried down under N_2_ stream and resuspended in 500 μl of 2:1:1 MeOH:isopropanol:hexane and filtered through 0.45 μm PTFE filters for analysis *via* RP LC-MS.

Core lipids and compounds released from methyl-iodide treatment were analyzed on an Agilent 1260 Infinity II series high-performance liquid chromatography instrument coupled to an Agilent G6125B single quadrupole mass spectrometer. Samples were ionized with electrospray ionization and detected in positive mode with the following parameters: 8.0 l/min drying gas flow rate, 35 psi nebulizer pressure, 3000 V capillary voltage, and either a 300 °C drying gas temperature (for core lipids) or 250 °C drying gas temperature (for methylthio-archaeol analysis). Core lipids were separated on a Kinetex 1.7 μm XB-C18 100 Å LC column (150 × 2.1 mm), and lipids released from methyl iodide treated lipoproteins were separated on an Agilent Poroshell 120 EC-C18 column (1.9 μm, 2.1 × 150 mm) as described in our previous work (43). Samples were analyzed in full scan mode with a m/z range of 600 to 750. To determine the relative abundance of each core lipid species, the m/z peak areas corresponding to the [M  +  H]^+^, [M + NH_4_]^+^, [M + Na]^+^, [M  +  K]^+^, and [M + Isopropanol + H]^+^ adduct ions were calculated and summed using Agilent MassHunter B.08.00 and verified manually. For archaeol, the adduct ions’ m/z values analyzed were as follows: [M  +  H]^+^ = 653.7, [M + NH_4_]^+^ = 670.8, [M + Na]^+^ = 675.7, [M  +  K]^+^ = 691.7, and [M + Isopropanol + H]^+^ = 712.8. For the unsaturated archaeols, the adduct ion m/z values analyzed were equal to the values for archaeol – (2 × number of double bonds). For quantification of the mass of methylthio-archaeol released from each sample, the peak areas of m/z = 683.7, 700.8, and 705.7 (corresponding to the [M + H]^+^, [M + NH_4_]^+^, [M + Na]^+^ adducts of methylthio-archaeol) were calculated and summed using Agilent MassHunter B.08.00 and inspected manually to confirm the proper integration of extracted ion peaks. The peak area was converted to the mass of methylthio-archaeol injected by the use of a 10-point standard curve as described in our previous work. The methylthio-archaeol (diphytanylglyceryl methyl thioether) standard was synthesized by Pharmaron, Inc. *via* a synthesis scheme previously described in Hong *et al.* (2025) (43).

### Quantitative PCR analysis of *aliA* expression

Three biological replicates of Δ*aliB* and wild type were collected at OD_600_ = 0.8 for each comparison, with 0.5 ml liquid cultures of each strain harvested and pelleted at 4900×*g* for 6 min. RNA was extracted using the Qiagen RNeasy Plus Micro Kit (Qiagen) on the QIAcube Connect automated platform (Qiagen), with lysates homogenized *via* QIAshredder Columns (Qiagen). RNA extracts were subjected to DNase digestion using the RNase-Free DNase kit (Qiagen) and further purified by RNeasy MinElute Cleanup Kit (Qiagen). RNA concentrations were measured with Qubit RNA Broad Range Assay Kits (Invitrogen), and integrity assessed using RNA ScreenTape on a 4200 Tapestation (Agilent). All samples had RINe values between 8.4 and 8.8. cDNA was synthesized from 1 μg RNA using the ProtoScript II First Strand cDNA Synthesis Kit (New England BioLabs) with random primers; parallel negative controls without reverse transcriptase (-RT) were prepared. Primers for *Hfx. volcanii* gene *aliA* (Table S5) were designed using Primer3 (85) and synthesized as RxnReady primers (IDT). Primers for the internal reference gene *eif1a* from prior studies were used (86). qRT PCR reactions (20 μl) contained 2× iTaq Universal SYBR Green Supermix (Bio-Rad), 5% DMSO, 300 nM primers, and 8 μl of 1:25 diluted cDNA. No-template (NTC) and -RT controls were included; reactions were considered acceptable if ΔCt ≤10 between sample and both controls. Efficiencies for *aliA* and *eif1a* primer sets were determined *via* standard curves and were 93.0% and 88.7% respectively. Each reaction included three biological replicates, each with three technical replicates, run on a QuantStudio 3 Real-Time PCR System (Applied Biosystems). Thermal cycling conditions were: 95 °C for 2 min, then 40 cycles of 95 °C for 15 s and 60 °C for 30 s. SYBR Green fluorescence (Rox normalized) was recorded at each cycle’s end, and the default melt curve program following the qPCR run verified primer specificity. Relative expression was calculated in Excel using the Pfaffl method (87), normalizing to *eif1a*, and compared to wild-type controls to determine differential expression.

### Immunoblotting

A single colony was used to inoculate a 5 ml liquid culture. At an OD_600_ of 0.75 to 0.80, cells from 4 ml liquid culture were harvested by centrifugation at 4300×*g* for 10 min at 4 °C. Cell pellets were then resuspended in 200 μl PBS buffer with 1 mM AEBSF and lysed by freezing (with liquid nitrogen or ethanol with dry ice) and thawing (at 37 °C) four times. 20 μl RQ1 RNase-Free DNase (Promega) was added to cell lysates, and the mixture was incubated at 37 °C for 30 min to degrade DNA. Unlysed cells were removed by centrifugation at 4300×*g* for 10 min at 4 °C, and the supernatant was transferred to a new tube for subsequent fractionation. To fractionate the lysates, 200 μl of cleared supernatant was subjected to ultracentrifugation at 81,000 rpm (231,472 × *g*) for 30 min at 4 °C using a TLA-100.2 fixed-angle rotor in a Beckman Coulter Optima MAX-TL ultracentrifuge. The resulting supernatant (cytoplasmic fraction) was transferred to a new ultracentrifuge tube, and the pellet (membrane fraction) was resuspended in 200 μl ice-cold PBS with 1 mM AEBSF. Both fractions were centrifuged again at 81,000 rpm for 30 min at 4 °C. Pellets were then resuspended in 200 μl PBS with 1 mM AEBSF, while the cytoplasmic fraction was transferred to a new tube. Maintaining identical final volumes ensured that each fraction represented an equivalent proportion of the starting cell lysate, permitting direct comparison of membrane and cytoplasmic fractions by loading equal sample volumes for Western blot analysis.

10 μl of HVO_1705 samples and 21 μl of HVO_0844 samples were supplemented with 50 mM dithiothreitol and 1x NuPAGE LDS Sample Buffer. PBS was added to adjust each sample to a final volume of 30 μl. Samples were incubated at 70 °C for 10 min, after which 27 μl of each sample was loaded onto NuPAGE 10%, Bis-Tris mini gels (8 cm × 8 cm, 1.0 mm × 10 wells) with NuPAGE MOPS SDS Running Buffer. After electrophoresis, proteins were transferred to a polyvinylidene difluoride (PVDF) membrane (Millipore) using a semi-dry transfer apparatus (BioRad) at 15 V for 30 min. Subsequently, the membrane was stained with Ponceau S Staining Solution (Cell Signaling Technology) to verify the successful protein transfer. The membrane was then washed for 10 min twice in PBS buffer and blocked for 1 h in 5% non-fat milk (LabScientific) in PBST buffer (PBS with 1% Tween-20). After blocking, the membrane was washed twice in PBST and once in PBS. For detection of the Myc tag, anti-C-Myc-9E10 (IgG1) antibody (UPenn Cell Center Service) was diluted 1000 times with PBS buffer containing 3% bovine serum albumin, which was then used to incubate the membrane overnight at 4 °C. The anti-C-Myc-9E10 (IgG1) antibody was validated by the manufacturer for Western blot detection of c-Myc–tagged proteins, as described in the product documentation, at a recommended dilution of 1:5000. Subsequently, the membrane was washed twice in PBST and once in PBS, followed by a 45-min incubation at room temperature with the secondary antibody solution (Amersham ECL anti-mouse IgG, horseradish peroxidase-linked whole antibody, from sheep (Cytiva) diluted 10,000 times in PBS containing 10% non-fat milk). After incubation, the membrane was washed three times in PBST and once in PBS. HRP activity was assessed using the Amersham ECL Prime Western Blotting Detection Reagent (GE). Membranes were imaged using an Amersham Imager 600, with image analysis conducted using the Amersham Imager 600 Analysis software. The results are representative of two independent experiments.

## Data availability

The LC-MS raw data and quantification for lipid analysis can be accessed through the Stanford Digital Repository [https://purl.stanford.edu/nk164tn8116]. The MS raw files generated for the proteomic analysis in this study have been deposited to the ProteomeXchange Consortium *via* the PRIDE partner repository with the dataset identifier PXD074718, and all identification and quantification results have been included in the deposited dataset. Raw and processed qPCR data are available under the identifier https://doi.org/10.5281/zenodo.20998375.

All other data supporting the findings of this study are provided within the paper and its supplementary information files. Source data are provided with this paper.

All scripts for the identification and quantification of peptides and proteins from MS datasets are made available through the Archaeal Proteome Project (http://archaealproteomeproject.org) and its associated GitHub repository (https://github.com/arcpp/ArcPP v1.4.0). Custom Python pipelines used for data filtering and figure generation are available at Zenodo under the identifier https://doi.org/10.5281/zenodo.20998375.

## Supporting information

This article contains supporting information (4, 42, 43, 55, 56, 88, 89).

## Conflict of interest

The authors declare that they have no conflicts of interest with the contents of this article.
