# Supplementary material for: Characterization of aliA and aliB deletion mutants reveals a dominant role of AliA in Haloferax volcanii lipoprotein lipidation
Source: J Biol Chem. 2026 Aug 25;302(10):113477. doi: 10.1016/j.jbc.2026.113477 (PMC13631571; doi:10.1016/j.jbc.2026.113477)
Supplement: Supplementary file 1 [file mmc1.docx]

**Supporting Information for**

Characterization of *aliA* and *aliB* deletion mutants reveals a dominant role of AliA in *Haloferax volcanii* lipoprotein lipidation

Yirui Hong^1^, Andy Garcia^2^, Samuel Kopelev^3^, Jacob A. Cote^1^, Friedhelm Pfeiffer^4^, Paula Welander^2^, Stefan Schulze^3*^, and Mechthild Pohlschroder^1*^

^1^Department of Biology, University of Pennsylvania, Philadelphia, Pennsylvania 19104, USA

^2^Department of Earth System Science, Stanford University, Stanford, California 94305, USA

^3^Thomas H. Gosnell School of Life Sciences, College of Science, Rochester Institute of Technology, Rochester, New York 14623, USA

^4^Computational Systems Biochemistry, Max Planck Institute of Biochemistry, 82152 Martinsried, Germany

***Corresponding author:** Mechthild Pohlschroder; Stefan Schulze

**Corresponding author email:** pohlschr@sas.upenn.edu; stefan.schulze@rit.edu

**This PDF file includes:**

Figures S1-S5

Table S1-S7

**Additional supporting materials for this manuscript are provided as separate files, as follows:**

Data S1 to S12 (1–3)

Source Data (original uncropped Western blot images used in Fig. 4 and the corresponding Ponceau S staining images)

**FIGURES**

**Fig. S1. Proteome coverage comparison between this study and datasets within the Archaeal Proteome Project.** Venn diagrams illustrating the overlap between **(a)** protein and **(b)** peptide identifications for this study and for the Archaeal Proteome Project (ArcPP, v. 1.4.0) (4). Bar plots showing the number of identified **(c)** proteins and **(d)** peptides for this study and for each dataset in the ArcPP. The same filtering criteria were applied for all datasets (≤1% PEP on the peptide spectrum match level (PSM), ≤1% FDR on the peptide level, ≤0.5% FDR on the protein level, and at least 2 PSMs for each level). Notably, out of the 30 proteins that were uniquely identified in this study, 18 are predicted to have ≥2 TM domains, and 2 are predicted to be processed by a prepilin peptidase. These results indicate that fractionation with Triton X-114 is beneficial for achieving high proteome coverage, including a high coverage of membrane proteins. The dataset with the second-highest number of protein identifications and highest number of peptide identifications (PXD021874), published later than the original ArcPP study, used subcellular fractionation (cytosol, membrane, and culture supernatant) and two different enzymes for protein digestion (trypsin and GluC) (5). It should also be noted that an additional large-scale proteomics study (6) was published while this manuscript was in preparation, achieving the highest protein and peptide coverage to date using a different protein extraction and mass spectrometry workflow; the implementation of this recent dataset into the ArcPP is pending.

 **Fig. S2. Heatmap of protein intensities for the 54 validated lipoproteins identified in Fig. 3a.** Protein intensities were z-score transformed across samples and are reflected by the color scale. Depicted lipoproteins were enriched in wild-type TX phase and exhibited at least a 4-fold reduction in TX phase abundance in ∆*aliA*/∆*aliB*, ∆*aliA*, or ∆*aliB* compared to the wild type, but were not significantly decreased in the corresponding AQ phase. Proteins were sorted by protein ID and by the mutant strains in which their abundance has been affected: (i) ∆*aliA*; (ii) ∆*aliA*/∆*aliB*; (iii) ∆*aliA* and ∆*aliB* and ∆*aliA*/∆*aliB*; all other proteins were affected in both ∆*aliA* and ∆*aliA*/∆*aliB*.

 **Fig. S3. Heatmap of protein intensities for the nine** **validated lipoproteins shown in Fig. 3b.** Protein intensities were z-score transformed across samples and are reflected by the color scale. Depicted lipoproteins were enriched in wild-type TX phase and exhibited a reduced TX phase abundance in ∆*aliA*/∆*aliB*, ∆*aliA*, or ∆*aliB* compared to the wild type. In contrast to Fig. S2, proteins were included here that were not quantified in the wild-type AQ phase or the mutant TX phase. Proteins were sorted by protein ID and by the mutant strains in which their abundance has been affected: (i) ∆*aliA*; (ii) ∆*aliA*/∆*aliB*; all other proteins were affected in both ∆*aliA* and ∆*aliA*/∆*aliB*.

**Fig. S4. Quantitative PCR showed a slight but non-significant increase in *aliA* expression in ∆*aliB* relative to the wild type (WT) (p-value = 0.174).** Bar height represents the mean gene expression ratio normalized to an internal control gene. Error bars indicate standard deviation from three biological replicates. Statistical significance was assessed using a two-tailed unpaired t-test assuming unequal variances (Welch’s t-test) for comparison between the log-transformed gene expression ratios of wild type and ∆*aliB*.

**Fig. S5. Exploratory analysis of quantitative proteomics results in the AQ and TX phases of the WT, Δ*aliA*, Δ*aliB*, and Δ*aliA*/Δ*aliB* strains. (a)** Principal component analysis plot for all analyzed samples. Each point represents one biological replicate. Percentages indicate the variance explained by the corresponding principal components. **(b)** Pearson correlation analysis across all analyzed samples. The heatmap shows the Pearson correlation for the pairwise comparison of all samples, with warmer colors indicating higher correlation. Samples are ordered based on hierarchical clustering reflecting the overall proteome similarity of between the samples. Each sample is color-coded and was measured in biological triplicates. Pearson correlation coefficients were calculated taking all quantified proteins from WT, Δ*aliA*, Δ*aliB*, and Δ*aliA*/Δ*aliB* strains in the AQ and TX phases into account. It should be noted that the plot is mirrored along the diagonal because of a symmetric duplication of data points for the pairwise comparisons.

**Fig. S6. Schematic illustration of missing-value handling in the MSstats workflow.** Four representative peptide-level scenarios are shown. **(a)** All peptides (Pep1 - Pep3) are successfully quantified in all replicates (R1 - R3); no imputation is required. **(b)** Some peptides are missing in individual replicates, but all missing peptide values could be imputed by MSstats; thus, all peptides are successfully quantified. **(c)** One peptide is entirely missing across replicates, but other peptides of the same protein are successfully quantified, allowing for the imputation of the remaining peptide by MSstats; thus, quantitative values for all peptides are provided. **(d)** No peptides are quantified for the protein in any replicate of an experimental condition; imputation cannot be performed by MSstats, and thus, no quantitative values are provided on the peptide or protein level. **(e)** When two conditions are compared (e.g. TX vs. AQ phase, or mutant vs. wild type), the log_2_FC will result in real values with a corresponding standard error (SE) if the protein was quantified in both conditions. Most figures and analyses described in this study refer to these cases, i.e. log_2_FCs with real values. **(f)** When a protein is not quantified in one condition of a condition comparison, MSstats returns the corresponding log_2_FC as Inf (if the protein is missing in Condition 2) or -Inf (if the protein is missing in Condition 1) without a corresponding SE. These proteins could be strongly enriched in one phase, and thus missing in the other, or might be absent in the wild-type or mutant strains. This information could be biologically meaningful, in addition to real log_2_FCs and corresponding p-values. Therefore, we further analyzed and reported proteins with a log_2_FC of Inf/-Inf in Fig. 1A and Fig. 2A (“Only TX” and “Only AQ”), as well as in Fig. 3B. However, since a reliable p-value could not be calculated due to the lack of SE, these proteins were reported separately from proteins described in **(e)**.

**TABLES**

**Table S1. Protein counts by predicted group and status in the mass spectrometry analysis of detergent (TX) and aqueous (AQ) phase in wild-type *Hfx. volcanii* (strain H53).** The numbers of proteins that were quantified only in the TX or AQ phase, or enriched in either phase, or showing no significant difference between the phases are listed. “Total” corresponds to the numbers of proteins for each predicted group within the whole theoretical proteome. ≥2 TM, proteins with at least two predicted transmembrane domains; Cyt, cytoplasmic proteins; Lipo, lipoproteins.

| **Category** | **Total** | **Quantified** | **Only in TX** | **Enriched in TX** | **Only in AQ** | **Enriched in AQ** | **No significant difference** |
| --- | --- | --- | --- | --- | --- | --- | --- |
| **≥2 TM** | 784 | 384 | 147 | 200 | 5 | 5 | 27 |
| **Cyt** | 3112 | 2045 | 23 | 376 | 107 | 1271 | 265 |
| **Lipo** | 108 | 87 | 13 | 68 | 0 | 1 | 5 |

**Table S2. Counts of *Hfx. volcanii* lipoproteins by enrichment status across strains.** “Total” refers to the number of predicted lipoproteins within the whole theoretical proteome. “Quantified (all strains)” refers to the total number of lipoproteins quantified in all four strains, whereas “Quantified (this strain)” and “Not quantified (this strain)” refer to number of lipoproteins that were or were not quantified, respectively, in the indicated strain.

| **Strains** | **Total** | **Quantified (all strains)** | **Quantified (this strain)** | **Only in TX** | **Enriched in TX** | **Only in AQ** | **Enriched in AQ** | **No significant difference** | **Not quantified (this strain)** |
| --- | --- | --- | --- | --- | --- | --- | --- | --- | --- |
| **WT** | 108 | 88 | 87 | 13 | 68 | 0 | 1 | 5 | 1 |
| **∆*aliA*/∆*aliB*** | 108 | 88 | 83 | 8 | 34 | 2 | 11 | 28 | 5 |
| **∆*aliA*** | 108 | 88 | 87 | 9 | 31 | 5 | 8 | 34 | 1 |
| **∆*aliB*** | 108 | 88 | 88 | 17 | 64 | 0 | 0 | 7 | 0 |

**Table S3. Lipoproteins validated through their reduced TX-phase abundance in *ali* deletion strains.** Predicted lipoproteins were considered validated if they were enriched in the TX phase of wild type and showed at least a 4-fold reduction in TX phase abundance in ∆*aliA*/∆∆*aliB*, ∆*aliA*, or ∆*aliB* compared to the wild type, but were not significantly decreased in the corresponding AQ phase. Proteins are grouped based on whether they were quantified in all relevant conditions for comparison (Fig. 3a) or showed missing values in either the wild-type AQ phase or the mutant TX phase (Fig. 3b). HVO_B0034* and HVO_0066* were identified in both analyses but met the detection criteria in different mutant strains. HVO_B0034 met the criteria only in Δ*aliA*/Δ*aliB* in the Fig. 3a analysis and only in Δ*aliA* in the Fig. 3b analysis, whereas HVO_0066 showed the opposite pattern. An additional lipoprotein, HVO_1245, was also included in the table. Although it was not predicted as a lipoprotein by SignalP 6.0 and was therefore absent from our predicted lipoprotein list, it was previously validated experimentally and was further supported by the MS data generated here.

| **Proteins quantified in all relevant conditions (Fig. 3a)** | | |
| --- | --- | --- |
| **Category** | **Count** | **Proteins** |
| **Decreased in ∆*aliA* and ∆*aliA*/∆*aliB*** | 49 | HVO_0022, HVO_0062, HVO_0447, HVO_0530, HVO_0564, HVO_0628, HVO_0807, HVO_1007, HVO_1240, HVO_1241, HVO_1242, HVO_1244, HVO_1401, HVO_1477, HVO_1603, HVO_1705, HVO_1888, HVO_2094, HVO_2113, HVO_2126, HVO_2153, HVO_2316, HVO_2375, HVO_2397, HVO_2432, HVO_2607, HVO_2695, HVO_2798, HVO_A0148, HVO_A0181, HVO_A0283, HVO_A0362, HVO_A0380, HVO_A0428, HVO_A0611, HVO_A0619, HVO_A0620, HVO_A0623, HVO_A0627, HVO_B0021, HVO_B0047, HVO_B0082, HVO_B0093, HVO_B0106, HVO_B0138, HVO_B0217, HVO_B0292, HVO_B0328, HVO_C0075 |
| **Decreased in ∆*aliA* only** | 2 | HVO_0066, HVO_2651 |
| **Decreased in ∆*aliA*/∆*aliB* only** | 2 | HVO_B0034, HVO_B0318 |
| **Decreased in ∆*aliA*/∆*aliB*, ∆*aliA*, and ∆*aliB*** | 1 | HVO_0553 |
| **Proteins missing in either WT AQ phase or the mutant TX phase (Fig. 3b)** | | |
| **Category** | **Count** | **Proteins** |
| **Decreased in ∆*aliA* and ∆*aliA*/∆*aliB*** | 6 | HVO_1119, HVO_1144, HVO_2286, HVO_A0549, HVO_B0144, HVO_B0228 |
| **Decreased in ∆*aliA* only** | 2 | HVO_A0557, HVO_B0034* |
| **Decreased in ∆*aliA*/∆*aliB* only** | 1 | HVO_0066* |
| **Previously experimentally confirmed but not predicted as lipoproteins in this study** | | |
| **Category** | **Count** | **Proteins** |
| **Decreased in ∆*aliA* and ∆*aliA*/∆*aliB*** | **1** | HVO_1245 |

**Table S4. Summary of MS results for *Hfx. volcanii* ArtA and the nine known ArtA substrates.**

| **Category** | **Protein ID** | **MS results** |
| --- | --- | --- |
| ArtA | HVO_0915 | No significant abundance differences between wild-type and mutant strains |
| ArtA substrates | HVO_0405; HVO_1095; HVO_1110; HVO_2006; HVO_2072; HVO_2160 | No significant abundance differences between wild-type and mutant strains |
|  | HVO_A0263; HVO_B0206 | Not detected in the datasets |

**Table S5. Plasmids, strains, and primers used in this study.**

|  | **Name** | **Relevant characteristic(s)** | **Source of reference** |
| --- | --- | --- | --- |
| **Plasmids** | pYH7 | pTA963 carrying C-terminally Myc-tagged *hvo_1705* | (3) |
|  | pYH9 | pTA963 carrying C-terminally Myc-tagged *hvo_0844* | (3) |
| **Strains** | H53 | ∆*pyrE2*/∆*trpA* | (7) |
|  | YH_113 | H53 ∆*aliA* | (3) |
|  | YH_57 | H53 ∆*aliB* | (3) |
|  | YH_93 | H53 ∆*aliA*/∆*aliB* | (3) |
|  | YH_75 | H53 containing pYH7 | (3) |
|  | YH_162 | ∆*aliA* containing pYH7 | (3) |
|  | YH_83 | ∆*aliB* containing pYH7 | (3) |
|  | YH_176 | ∆*aliA*/∆*aliB* containing pYH7 | (3) |
|  | YH_74 | H53 containing pYH9 | (3) |
|  | YH_159 | ∆*aliA* containing pYH9 | (3) |
|  | YH_82 | ∆*aliB* containing pYH9 | (3) |
|  | YH_173 | ∆*aliA*/∆*aliB* containing pYH9 | (3) |
| **Primers** | qPCR_*aliA*_F | CAACGGTAGCAGAGCGAATG | This study |
|  | qPCR_*aliA*_R | TCCCCAGAAGTAGTGCAAGA | This study |

**Table S6. Instrument parameters and chromatography gradient that were used during the chromatographic separation and mass spectrometric analysis of proteomics samples.**

| **Category** | **Descriptions** |
| --- | --- |
| **Column** | nanoEase M/Z Peptide BEH C18 column, Waters Corporation, 1.7 um particle size, 75 um x 250 mm |
| **Column oven** | 50°C |
| **Flow rate** | 300 nl/min |
| **Buffer system** | Solvent A: 0.1% formic acid in H_2_O (Optima™ grade) |
|  | Solvent B: 0.1% formic acid in acetonitrile (Optima™ grade) |
| **Gradient** | 1 min 3% B; increase to 17% B over 39 min; increase to 32% B over 20 min; increase to 90% B over 2 min; 9 min 90% B; decrease to 3% B over 2 min; 17 min 34% B |
| **Mass spectrometry** | |
| **Ion mode** | positive |
| **Excluded charge states** | 1, >6, unknown |
| **Dynamic exclusion** | 10 sec, after 1 observation, tolerance window 10 ppm |
| **MS1 Resolution** | 120,000 |
| **MS1 Maximum injection time** | 50 ms |
| **MS1 AGC target** | 250% (1e6) |
| **MS1 Detector** | Orbitrap |
| **MS1 Scan range** | 400-2000 *m/z* |
| **MS2 Resolution** | 15,000 |
| **MS2 Detector** | Orbitrap |
| **MS2 AGC target** | 200% (1e5) |
| **MS2 Maximum injection time** | dynamic |
| **MS2 Scan range** | automatic |
| **MS2 Data-dependent acquisition** | cycle time 3 s |
| **Fragmentation** | Higher-energy collisional dissociation |
| **MS2 Normalized collision energy** | stepped: 25, 30, 35 |

**Table S7. Parameters used for the bioinformatic processing of proteomics results. All parameters that are not listed were left at their default values.**

| **Ursgal (version 0.6.9): protein database search, statistical post-processing, peptide quantification** | |
| --- | --- |
| **enzyme** | trypsin (cleavage on C-terminal side of K and R, unless followed by P) |
| **precursor_mass_tolerance_minus,**  **precursor_mass_tolerance_plus** | 4 ppm |
| **frag_mass_tolerance** | 15 ppm |
| **max_missed_cleavages** | 2 |
| **modifications** | "C,fix,any,Carbamidomethyl",  "M,opt,any,Oxidation",  "*,opt,Prot-N-term,Acetyl" |
| **percolator_post_processing** | tdc |
| **csv_filter_rules** | ["combined PEP", "lte", 0.01],  ["Conflicting uparam", "contains_not", "enzyme"] |
| **isotopic_distribution_tolerance** | 5 |
| **normalize_intensities** | True |
| **match_between_runs** | True |
| **match_between_runs_RT_window** | 0.5 min |
| **use_shared_peptides** | True |
| **MSstats (version 4.0): differential abundance analysis** | |
| **MBimpute** | True |
| **logTrans** | 2 |
| **normalization** | equalizeMedians |

**References**

1.	Giménez, M. I., Dilks, K., and Pohlschröder, M. (2007) *Haloferax volcanii* twin-arginine translocation substates include secreted soluble, C-terminally anchored and lipoproteins. *Mol. Microbiol.* **66**, 1597–1606

2.	Storf, S., Pfeiffer, F., Dilks, K., Chen, Z. Q., Imam, S., and Pohlschröder, M. (2010) Mutational and bioinformatic analysis of haloarchaeal lipobox-containing proteins. *Archaea*. **2010**, 410975

3.	Hong, Y., Makarova, K. S., Garcia, A. A., Xu, R., Pfeiffer, F., Welander, P. V., and Pohlschroder, M. (2025) Uncovering the prevalence, key biogenesis enzymes, and biological significance of archaeal lipoproteins. *Nat. Commun.* **16**, 8411

4.	Schulze, S., Adams, Z., Cerletti, M., De Castro, R., Ferreira-Cerca, S., Fufezan, C., Giménez, M. I., Hippler, M., Jevtic, Z., Knüppel, R., Legerme, G., Lenz, C., Marchfelder, A., Maupin-Furlow, J., Paggi, R. A., Pfeiffer, F., Poetsch, A., Urlaub, H., and Pohlschroder, M. (2020) The Archaeal Proteome Project advances knowledge about archaeal cell biology through comprehensive proteomics. *Nat. Commun.* **11**, 3145

5.	Schulze, S., Pfeiffer, F., Garcia, B. A., and Pohlschroder, M. (2021) Comprehensive glycoproteomics shines new light on the complexity and extent of glycosylation in archaea. *PLoS Biol.* **19**, e3001277

6.	Franco, P. H. C., Üresin, D., Borst, A., Rusling, L. A., Soppa, J., and Langer, J. D. (2026) Proteome characterization of single gene deletion mutants lacking zinc finger µ-proteins in *Haloferax volcanii*. *microLife*. 10.1093/femsml/uqag005

7.	Allers, T., Ngo, H.-P., Mevarech, M., and Lloyd, R. G. (2004) Development of additional selectable markers for the halophilic archaeon *Haloferax volcanii* based on the *leuB* and *trpA* genes. *Appl. Environ. Microbiol.* **70**, 943–953
